# A Dual‐Adjuvanted Parenteral‐Intranasal Subunit Nanovaccine generates Robust Systemic and Mucosal Immunity Against SARS‐CoV‐2 in Mice

**DOI:** 10.1002/advs.202402792

**Published:** 2024-10-01

**Authors:** Bhawana Pandey, Zhengying Wang, Angela Jimenez, Eshant Bhatia, Ritika Jain, Alexander Beach, Drishti Maniar, Justin Hosten, Laura O'Farrell, Casey Vantucci, David Hur, Richard Noel, Rachel Ringquist, Clinton Smith, Miguel A. Ochoa, Krishnendu Roy

**Affiliations:** ^1^ Wallace H. Coulter Department of Biomedical Engineering Georgia Institute of Technology Atlanta GA USA; ^2^ Woodruff School of Mechanical Engineering Georgia Institute of Technology Atlanta GA USA; ^3^ Physiological Research Laboratory Georgia Institute of Technology Atlanta GA USA; ^4^ The Parker H. Petit Institute for Bioengineering and Biosciences School of Chemical & Biomolecular Engineering Georgia Institute of Technology Atlanta GA USA; ^5^ Wallace H. Coulter Department of Biomedical Engineering The Parker H. Petit Institute for Bioengineering and Biosciences Marcus Center for Therapeutic Cell Characterization and Manufacturing Georgia Institute of Technology Atlanta GA USA; ^6^ Department of Biomedical Engineering Department of Chemical and Biomolecular Engineering School of Engineering Department of Pathology, Microbiology and Immunology School of Medicine Vanderbilt University Nashville TN USA

**Keywords:** antiviral immunity, combination adjuvants, mucosal immunity, parenteral and intranasal vaccination, polymer nanoparticles, SARS‐CoV‐2 subunit nanovaccine

## Abstract

Existing parenteral SARS‐CoV‐2 vaccines produce only limited mucosal responses, essential for reducing transmission and achieving sterilizing immunity. Appropriately designed mucosal boosters can overcome the shortcomings of parenteral vaccines and enhance pre‐existing systemic immunity. Here, a new protein subunit nanovaccine is developed by utilizing dual‐adjuvanted (RIG‐I: PUUC RNA and TLR‐9: CpG DNA) polysaccharide‐amino acid‐lipid nanoparticles (PAL‐NPs) along with SARS‐CoV‐2 S1 trimer protein, that can be delivered both intramuscularly (IM) and intranasally (IN) to generate balanced mucosal‐systemic SARS‐CoV‐2 immunity. Mice receiving IM‐Prime PUUC+CpG PAL subunit nanovaccine, followed by an IN‐Boost, developed high levels of IgA, IgG, and cellular immunity in the lungs and showed robust systemic humoral immunity. Interestingly, as a purely intranasal subunit vaccine (IN‐Prime/IN‐Boost), PUUC+CpG PAL‐NPs induced stronger lung‐specific T cell immunity than IM‐Prime/IN‐Boost, and a comparable IgA and neutralizing antibodies, although with a lower systemic antibody response, indicating that a fully mucosal delivery route for SARS‐CoV‐2 vaccination may also be feasible. The data suggest that PUUC+CpG PAL subunit nanovaccine is a promising candidate for generating SARS‐CoV‐2 specific mucosal immunity.

## Graphical abstract

A SARS‐CoV‐2 subunit nanovaccine is developed utilizing dual‐adjuvanted PAL nanoparticles, which can be administered via both parenteral and intranasal routes. This nanovaccine, when given intramuscular prime and mucosal boost, generates strong mucosal and cellular lung immunity coupled with robust systemic humoral response. These findings highlight the potential of combined systemic and mucosal vaccination to achieve sterilizing immunity against COVID‐19.

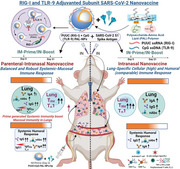

## 1 Introduction

The COVID‐19 pandemic continues to cause a global health crisis with frequent viral mutations and uncontrolled transmission in many parts of the world. In the U.S. alone, monthly death rates from COVID‐19 remained well over 1000, at last reporting in February 2024, even 4 years after the start of the pandemic.^[^ ^1^ ^]^ Almost 72% of the world's population have received the licensed parenteral mRNA‐LNP and/or adenoviral vectors‐based vaccines.^[^ ^1^, ^2^, ^3^ ^]^ These approved vaccines are designed to be administered via intramuscular (IM) route, show high efficacy against severe SARS‐CoV‐2 infections, and reduce hospitalization and deaths.^[^ ^2^, ^3^ ^]^ Unfortunately, recent studies have shown that even after vaccination with booster doses, there are asymptomatic, symptomatic, and some severe cases of SARS‐CoV‐2 infection observed after a few months. These results indicate declining antiviral immunity within a short period and raise questions about durable efficacy and protection through these vaccines.^[^ ^4^, ^5^ ^]^ The limited efficacy of approved vaccines is due to several factors, including poor respiratory mucosal immunity, viral immune evasion (continuous viral mutation), and increased viral transmission.^[^ ^4^, ^5^, ^6^ ^]^ Additionally, vaccine accessibility and acceptance remain significant concerns.^[^ ^5^ ^]^

SARS‐CoV‐2 is primarily a respiratory pathogen.^[^ ^7^ ^]^ IM vaccinations predominantly induce systemic immune responses (circulating antibodies, memory B cells, effector T cells), with limited mucosal immunity at the sites of infection, i.e., nasopharynx and lungs. IM vaccines alone leave the upper respiratory tract vulnerable to viral replication and dissemination, leading to reduced sterilizing immunity.^[^ ^8^, ^9^ ^]^ Combining IM with mucosal vaccination could generate both mucosal and systemic antiviral immune responses similar to natural infection and may ultimately lead to better protection and reduced transmission.^[^ ^10^, ^11^, ^12^, ^13^ ^]^ Antiviral mucosal immunity is characterized by the generation of robust mucosal IgA, IgG, and neutralizing antibodies (nAbs) in the nasopharynx and the lungs, as well as lung‐specific cellular immunity (Tissue‐resident memory T and B cells) and systemic immune responses.^[^ ^10^, ^11^, ^13^, ^14^, ^15^ ^]^

Vaccines which generate mucosal immunity can be used as a booster for the already IM‐vaccinated population (heterologous IM‐Prime/IN‐Boost) or can be employed for both priming and boosting (homologous IM‐Prime/IN‐Boost and IN‐Prime/IN‐Boost) in the unvaccinated population.^[^ ^8^, ^16^, ^17^, ^18^, ^19^ ^]^ The IN‐boosting in IM‐Prime/IN‐Boost route (both hetero and homologous) could strengthen pre‐existing circulating immunity achieved via intramuscular priming (a prime‐pull mechanism), whereas IN‐Prime/IN‐Boost approach could increase acceptance. Recently, encouraging mucosal vaccine‐based preclinical findings have emerged utilizing different prime‐boost approaches. For example, IN‐boost of adenoviral vector vaccines shows a higher response, but the use of viral vector vaccines has raised safety and efficacy concerns,^[^ ^8^ ^]^ whereas the use of mRNA‐LNP vaccines for mucosal boosting encountered issues related to dose‐limiting toxicity.^[^ ^8^, ^15^, ^16^ ^]^ In contrast, the improved design of the polymeric mRNA‐NP vaccine exhibited effective protection through mucosal boosting alone,^[^ ^16^ ^]^ rather than through both the mucosal‐Prime/Boost methods,^[^ ^17^ ^]^ a situation that could potentially benefit from the incorporation of adjuvants.^[^ ^19^ ^]^ Alternative attempts to use adjuvanted subunit vaccines, which involve the direct administration of adjuvants alongside subunit antigens such as spike trimer,^[^ ^8^ ^]^ or recombinant toxin‐conjugated antigen,^[^ ^18^ ^]^ have faced challenges in achieving desired outcomes, which might be due to the absence of appropriate adjuvant delivery systems. To bridge these gaps, we explored an innovative protein subunit mucosal nanovaccine incorporating a combination of adjuvants, aimed at providing robust and lasting mucosal immunity.

Protein subunit vaccines are known to be comparatively safer as they use only a fragment of a pathogen,^[^ ^20^ ^]^ and require immune adjuvants to boost their potency.^[^ ^20^, ^21^, ^22^ ^]^ Combination's adjuvants on biomaterial‐based polymeric nanovaccines increase antigen immunogenicity by activating multiple pattern recognition receptors (PRRs) of the innate immune system, modulating the antigen pharmacokinetics, and facilitating antigen dose sparing.^[^ ^20^, ^21^, ^22^ ^]^ Few adjuvants that target multiple PRRs, including RLRs (Retinoic Inducible Gene 1: (RIG‐I)‐Like Receptors) and Toll‐Like Receptors (TLRs: 4, 7/8, 9), either alone or in combination, have been explored for SARS‐CoV‐2 mucosal vaccines, but are not able to provide complete protection, thus required more research and investigation.^[^ ^8^, ^18^, ^23^, ^24^, ^25^ ^]^

As of March 2023, over a dozen mucosal vaccines are in clinical trials, and five of them have been authorized for use or registered for regulatory agency review for SARS‐CoV‐2. Out of these five, three are viral vector‐based vaccines: Bharat Biotech in India, Gamaleya in Russia, and CanSino Biologics in China.^[^ ^12^, ^13^ ^]^ None of these vaccines have been authorized for use in the United States or Europe, nor has the WHO granted them an emergency use listing. Mucosal vaccines for influenza that utilized intranasal live attenuated pathogens had been approved previously but were not fully effective and safe.^[^ ^26^ ^]^ Therefore, there is a need to develop new mucosal vaccine strategies that not only overcome the limitations of IM vaccines and achieve high levels of durable protection against infection but also mitigate the post‐COVID severe effects, such as post‐acute sequelae of SARS‐CoV‐2 infection and long COVID.

Here, we designed a novel, dual‐adjuvanted protein subunit SARS‐CoV‐2 nanovaccine formulation by exploring different adjuvants as PRR agonists‐ PUUC RNA: RLRs agonist, CpG DNA: TLR‐9 agonist and R848: TLR‐7/8 agonist, which are loaded on degradable, polysaccharide‐amino acid‐lipid polymeric nanoparticles (PAL‐NPs). To study the adjuvant‐mediated SARS‐CoV‐2 immune responses through (IM) prime and intranasal (IN) boost strategy, we explored multiple adjuvants, both individually and in combinations, incorporated into PAL‐NPs. The primary goal was to identify the best combination adjuvant formulation that would potentially enhance both mucosal and systemic immunity against SARS‐CoV‐2. We further proceeded with a comprehensive investigation of immunization route‐specific immune responses using the promising dual‐adjuvant nanovaccine candidate. Different prime‐boost approaches were explored to evaluate their effectiveness in generating a strong, balanced, and lung‐specific mucosal and systemic immune response.

## 2 Results

### 2.1 Synthesis and Characterization of Adjuvanted PAL‐NP Subunit Vaccine Formulations

**Figure 1:**
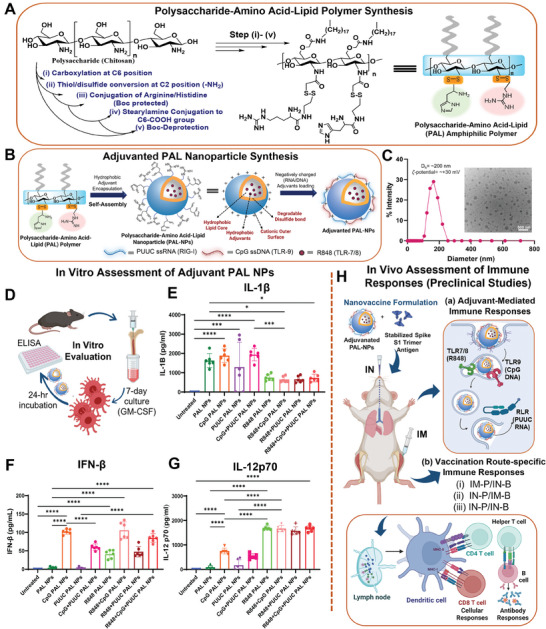
Synthesis and characterization of adjuvant loaded PAL‐NPs. A) Multistep synthetic scheme of polysaccharide‐amino acid‐lipid (PAL) amphiphilic polymer. B) Schematic of PAL‐NPs fabrication and adjuvants loading (encapsulated: R848 and surface‐loaded nucleic acids: PUUC RNA, CpG DNA). C) Physiochemical characterization of PAL‐NPs: hydrodynamic diameter and zeta potential (inset: TEM image, scale bar: 500 nm). D) Murine GM‐CSF differentiated Bone Marrow Dendritic Cells (mBMDCs) were treated with single/dual/triple adjuvanted PAL‐NP formulations and controls for 24 h. E–G) Analysis of proinflammatory cytokines: IL‐1β E), IFN‐β F), and IL12p70 G) after treatment of adjuvanted PAL‐NPs (n = 6) with differentiated mBMDCs. H) Designed in vivo studies for the assessment of adaptive immune responses (both lung‐specific and systemic) using adjuvant nanovaccine formulations via two strategies: a) adjuvant‐mediated and b) different prime‐boost vaccination routes. Error bars represent SEM (standard error of the mean). Statistical significance was determined by one‐way ANOVA followed by Tukey's post‐hoc test for multiple comparisons. *p ≤ 0.05, **p ≤ 0.01, ***p ≤ 0.001, ****p ≤ 0.0001 for all graphs.

We developed a dual‐adjuvanted nanoparticle‐based SARS‐CoV‐2 protein subunit vaccine using a newly designed synthetic polymer‐lipid nanoparticle and S1 trimer subunit protein. This polymer‐lipid conjugate macromolecule enhanced the combination adjuvant delivery, thereby facilitating the nanovaccines ability to generate a strong and balanced mucosal‐systemic SARS‐CoV‐2 immunity (**Figure** **1**). Combination adjuvant delivery can be enhanced by controlling polymeric nanoparticle's physiochemical properties, which depend on the appropriate selection and design of polymers.^[^ ^27^ ^]^ Therefore, we designed and synthesized the amphiphilic polymer‐lipid [OCMC‐S‐S‐(A/H)‐SA], namely‐ polysaccharide‐amino acid‐lipid (PAL polymer), by chemical modification of polysaccharide (chitosan Mw: 15 KDa) backbone with stearyl group (lipid) at C6 position and amino acids (Arginine and Histidine), and disulfide linker at C2 position (Figure 1A; Figure S1A, Supporting Information). Details of polymer synthesis and characterization methods can be found in Supporting Information (Materials and Methods). Cationic and biodegradable PAL‐NPs were fabricated via self‐assembly of amphiphilic PAL polymer using the probe sonication method. Further, pathogen‐mimicking PAL‐NPs were prepared by the loading of different adjuvant combinations through surface electrostatic adsorption of anionic adjuvants: PUUC RNA (target cytoplasmic RIG‐I‐like receptors: retinoic acid‐inducible gene‐I) and CpG DNA (target endosomal TLR‐9 receptors), and by encapsulation of hydrophobic adjuvants (R848: target endosomal TLR‐7/8) (Figure 1B; Table S1, Supporting Information). The blank and R848 PAL‐NPs were ≈100–200 nm in diameter (Figure 1C; Table S1, Supporting Information). Before surface loading, the zeta potential of PAL‐NPs was ≈+30 mV at pH ≈7.0, making it appropriate for loading nucleic acid adjuvants (Figure 1C).^[^ ^28^, ^29^, ^30^ ^]^ The surface loading of CpG and PUUC on the PAL‐NP surface resulted in some aggregation, increasing the average hydrodynamic size to ≈200–300 nm, whereas the zeta potential was reduced to ≈+11 mV, which is consistent with previous results.^[^ ^28^, ^29^, ^30^ ^]^ Transmission electron microscopy (TEM) was used to determine the morphology of PAL‐NPs, revealing a well‐dispersed spherical shape with an average diameter between 150 and 200 nm (Figure 1C, inset). The degradability of PAL‐NPs mediated by disulfide bond reduction was confirmed by a decrease in nanoparticle size from ≈200 to ≈100 nm or below in the presence of dithiothreitol (DTT) for 24 h (Figure S1C, Supporting Information), which was not observed in disulfide‐bond deficient PAL‐NPs (non‐degradable PAL‐NPs).

The modified polysaccharide‐amino acid polymer showed an extended buffering range (pH 5.5‐8) compared to the polysaccharide alone (Figure S1D, Supporting Information), indicating enhanced buffering capacity due to arginine and histidine modification. PUUC adjuvant release was notably increased under low pH and in the presence of DTT, which confirms the dual‐responsive behavior of PAL‐NPs (Figure S1E, Supporting Information). No significant changes were noted in these physiochemical parameters (size and zeta potential) of PAL‐NPs over four weeks in PBS (pH = 7.2), confirming their stability (Figure S1F, Supporting Information). The encapsulated R848 was also retained in the PAL‐NP core after lyophilization and up to 1 month, which confirms that PAL‐NPs maintain structural integrity. Monitoring the size and zeta potential of lyophilized PAL‐NPs over a month revealed no significant changes, although a slight increase in nanoparticle aggregation was observed (Figure S1G, Supporting Information).

To examine the immunostimulatory effects of different adjuvants and their combinations on PAL‐NPs in vitro, we screened eight PAL‐NPs adjuvant formulations (CpG, PUUC, CpG+PUUC, R848, R848+CpG, R848+PUUC, R848+CpG+PUUC) (Figure 1D–F). These formulations were incubated with GM‐CSF‐derived murine bone‐marrow‐derived dendritic cells (mBMDCs) for 24 h, and collected supernatants were analyzed to quantify proinflammatory cytokine secretion (Table S1, Supporting Information). PUUC+CpG PAL‐NPs significantly increased the secretion of proinflammatory cytokine IL‐1β, but its secretion was mainly driven by CpG (Figure 1E). R848+CpG PAL‐NPs synergistically increased the IFN‐β secretion (Figure 1F). R848 PAL‐NPs is the only group that significantly stimulated the secretion of IL12p70 (Figure 1G). Surprisingly, the blank PAL‐NPs, which were considered a control group due to the absence of real adjuvants, also generated considerable IL‐1β secretion but did not stimulate IFN‐β and IL12p70 secretion. However, adjuvant combinations formulations of PUUC+CpG and R848+CpG, as well as CpG alone on PAL‐NPs, enhanced the stimulation of the initial innate immune responses through RLRs (PUUC) and TLRs (CpG: TLR‐9 and R848: TLR‐7/8) signaling (Figure 1H). These results inspired us to evaluate whether these single and dual adjuvanted PAL‐NPs would enhance the SARS‐CoV‐2 immune responses in vivo (Figure 1H).

### 2.2 PUUC+CpG PAL‐NPs Protein Subunit Vaccine Elicits Robust SARS‐CoV‐2 Mucosal and Systemic Humoral Immunity, when Delivered IM‐Prime/IN‐Boost

**Figure 2:**
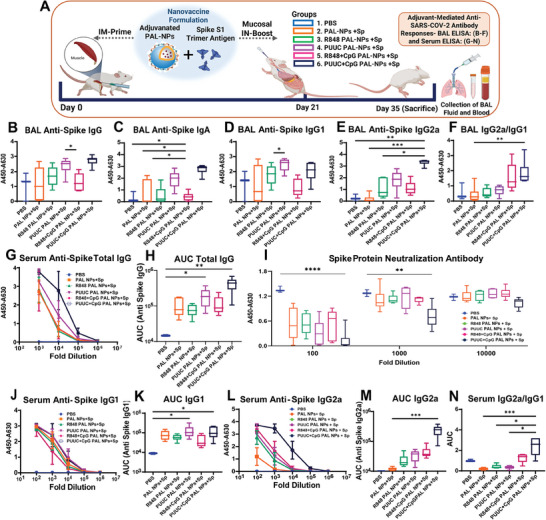
A dual‐adjuvanated PAL‐NPs (PUUC+CpG) subunit nanovaccine elicits robust SARS‐CoV‐2 mucosal and systemic humoral immunity, when delivered IM‐Prime/IN‐Boost. A) Vaccination study design: Female BALB/c mice (n=3 for PBS and n=6 for other PAL‐NP formulations) were immunized IM at day 0 (1st dose) with nanovaccine formulation of adjuvanted PAL‐NPs (NPs: 250 µg, PUUC: 20 µg, CpG: 40 µg and R848: 20 µg) and stabilized spike (Sp) S1 trimer protein at a dose of 1 µg respectively. On day 21, mice received the 2nd dose of vaccine formulation IN using similar doses of adjuvants, PAL‐NPs, and spike protein, except for the CpG dose, which was reduced to 20 µg. Mice were euthanized after 2 weeks on day 35 to collect BAL fluid and serum. BAL fluid and serum from vaccinated mice were assayed with ELISA assay. B–E) BAL fluid from vaccinated mice was assayed for anti‐spike IgG B), IgA C), IgG1 D), and IgG2a E) with ELISA at 1:5 dilution. F) Quantification of BAL IgG2a/IgG1. G,H) Anti‐spike total IgG in serum at various dilutions measured by absorbance (A450‐A630 nm) and comparison of area under the curve (AUC). I) ACE‐2 signal measured by absorbance (A450‐A630 nm) in spike protein neutralization assay with ELISA. Lower absorbance values indicate higher spike‐neutralizing antibody levels in serum. J,K) Quantification of serum anti‐spike IgG1 and comparison of AUC. L,M) Quantification of serum anti‐spike IgG2a and comparison of AUC. N) Quantification of serum IgG2a/IgG1 ratio. Error bars represent the SEM. Normality was assessed with the Kolmogorov–Smirnov test. Statistical significance was determined with the Kruskal–Wallis test and Dunn's post‐hoc test for multiple comparisons. *p ≤ 0.05, **p ≤ 0.01, ***p ≤ 0.001, ****p ≤ 0.0001 for all graphs.

Four selected adjuvanted PAL‐NPs (PUUC, R848, R848+CpG, PUUC+CpG) vaccine formulations were screened in vivo using IM‐Prime/IN‐Boost immunization strategy. Through this study, we evaluated the best adjuvant combination on PAL‐NPs that elicit both mucosal and systemic humoral immune responses in a balanced and potent manner (**Figure** **2**A). The adjuvanted nanovaccine formulations were prepared by combining adjuvanted PAL‐NPs with recombinant and stabilized SARS‐CoV‐2 S1 trimer subunit as the target antigen. The S1 trimer subunit is more immunogenic than the RBD alone due to the presence of other spatial epitopes along with RBD, which contribute to virus neutralization and maintain the natural configuration of antigen.^[^ ^31^ ^]^ Mice were vaccinated IM‐Prime at day 0 and boosted via IN route at day 21 with adjuvanted PAL‐NPs vaccine formulations and controls. Mice were sacrificed at day 35 (14 days post‐boost), and BAL (bronchoalveolar lavage) fluid and serum samples were collected to analyze the generated humoral response. The blank NPs exhibited minimal immune responses; therefore, we considered them as control group along with PBS. We found that PUUC+CpG PAL‐NP group significantly and robustly increased BAL anti‐spike IgG and IgA levels at 1:5 dilution compared to other formulations, including control groups (Figure 2B,C). We assessed T_H_1/T_H_2 skewed IgG (IgG1 and IgG2a subsets) antibody responses in BAL fluid elicited after adjuvanted PAL‐NP immunization, revealing a significantly higher level of T_H_1‐associated IgG2a response in mice with PUUC+CpG PAL‐NP vaccine formulation (Figure 2D–F).

Further, we assessed systemic IgG and nAbs responses in serum (Figure 2G–I). The PUUC+CpG PAL‐NPs group significantly increased the levels of anti‐SARS‐CoV‐2 IgG Abs in serum at 10^5^‐fold dilution (Figure 2G). The generated serum IgG levels (calculated with area under the curve: AUC) after administration of adjuvanted nanovaccine formulation and control groups followed this order: PUUC+CpG>PUUC>R848+CpG>R848 = PAL‐NPs (Figure 2H). nAbs play a crucial role in reducing the replication of SARS‐CoV‐2 and are essential in protecting against severe infections caused by the virus.^[^ ^32^ ^]^ Thus, we assessed generated serum nAbs by measuring the inhibition of the spike RBD‐ACE2 interaction using an ELISA‐based ACE2 competition assay (Figure 2I). We found that PUUC+CpG PAL‐NPs group elicited high titers of nAbs, which is detectable at 10^4^‐fold dilution and also higher than the other groups. The complete neutralization was observed at 100‐fold dilution. We observed that both PUUC+CpG and PUUC groups (alone), elicited similar and higher levels of IgG1 (Figure 2J,K), whereas only PUUC+CpG PAL‐NP group significantly induced highest anti‐spike IgG2a titers (Figure 2L,M). The highest IgG2a/IgG1 ratio was observed in PUUC+CpG PAL‐NPs immunized mice, indicating a higher T_H_1‐biased humoral response (Figure 2N).

### 2.3 PUUC+CpG PAL‐NPs Protein Subunit Vaccine Induces Robust SARS‐CoV‐2 Mucosal T Cell and Antigen‐Specific B Cell Immune Responses, when Delivered IM‐Prime/IN‐Boost

**Figure 3:**
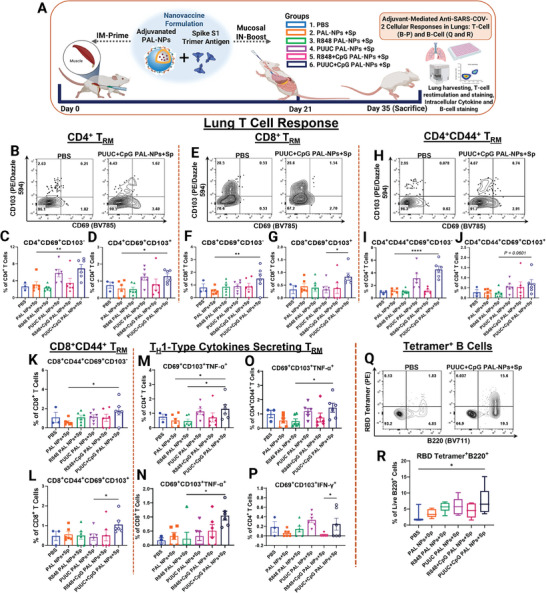
PUUC+CpG dual‐adjuvanted PAL‐NPs subunit nanovaccine elicits robust SARS‐CoV‐2 mucosal cellular immunity when delivered IM‐Prime/IN‐Boost. A) Vaccination study design: Female BALB/c mice were immunized on days 0 (IM prime) and 21 (IN boost) with adjuvanted PAL‐NP vaccine formulation combined with S1 spike protein (see Table S1, Supporting Information for doses). Harvested lung cells on day 35 were restimulated with spike peptide pools for 6 h and stained for analysis by flow cytometry. B–D) CD4^+^ T_RM_ flow cytometry (FCM) plots B), percentage of CD4^+^CD69^+^CD103^−^ C) and CD4^+^CD69^+^CD103^+^ cell populations D). E–G) CD8^+^ T_RM_ FCM plots E), percentage of CD8^+^CD69^+^CD103^−^ F) and CD8^+^CD69^+^CD103^+^ cell populations G). H–J) CD4^+^CD44^+^ T_RM_ FCM plots H), percentage of CD4^+^CD44^+^CD69^+^CD103^−^ I) and CD4^+^CD44^+^CD69^+^CD103^+^ J) cell populations. K,L) Percentage of CD8^+^CD44^+^CD69^+^CD103^−^ K) and CD8^+^CD44^+^CD69^+^CD103^+^ L) cell populations. M–O) Percentage of monofunctional CD4^+^ T_RM_ M), CD8^+^ T_RM_ N), and CD4^+^CD44^+^ T_RM_ O) cells expressing TNF‐α. P) Percentage of monofunctional CD4^+^T_RM_ cell population expressing IFN‐γ. Lung cells were stained for B cell markers and analyzed by flow cytometry. Q,R) Representative FCM plots and percentage of RBD tetramer^+^ B220 cells. Outliers were identified by the ROUT method and removed. Error bars represent the SEM. Statistical significance was calculated using one‐way ANOVA followed by Bonferroni's post‐hoc test for the figures (C–N) and Tukey's post‐hoc test for figures O) and R) for multiple comparisons. *p ≤ 0.05, **p ≤ 0.01, ***p ≤ 0.001, ****p ≤ 0.0001 for all graphs. ns represents the non‐significant values.

The adaptive cellular immune responses are generated and functional in the local tissues during respiratory infection and provide long‐lasting protective immunity at the infection sites.^[^ ^11^, ^14^, ^33^ ^]^ Traditionally, adjuvants are known for inducing and enhancing potent T cell responses in protein subunit vaccines.^[^ ^21^ ^]^ To investigate adjuvant‐mediated T cell responses in lungs, elicited through nanovaccine immunization on 35th day, the restimulated lung cells were stained with canonical T cell markers and analyzed with flow cytometry (**Figure** **3**A). The gating strategy for lung T cells subset is shown in Figure S8A (Supporting Information). PUUC+CpG PAL‐NP group exhibited a significant and higher frequency of CD4^+^ T cells that express both CD69^+^CD103^−^ (≈2.75 fold) (Figure 3B,C) as well as CD69^+^CD103^+^ markers (≈1.70 fold) (Figure 3B,D), compared to PBS control and was also highest among all other PAL‐NP groups. Similarly, lung CD8^+^ T cells significantly expressed higher levels of CD69^+^CD103^−^ (≈1.90 fold) (Figure 3E,F) and CD69^+^CD103^+^ markers (≈2.5 fold) (Figure 3E,G), after PUUC+CpG PAL‐NP vaccine immunization. The CD69 is the earliest activation marker generated on the surfaces of antigen‐specific activated lymphocytes.^[^ ^34^ ^]^ Memory CD4^+^ and CD8^+^ T cells are more prominent in the local tissues and non‐circulating, known as tissue‐resident memory (CD69^+^CD103^+^) T cells (T_RM_).^[^ ^35^ ^]^ We did not observe significant CD4^+^CD44^+^ and CD8^+^CD44^+^ T cell populations with any adjuvanted PAL‐NPs vaccine formulation (Figure S3A,E, Supporting Information). However, PUUC+CpG PAL‐NP formulation did induce effector resident memory CD4^+^ (Eff T_RM_) cell populations (CD4^+^CD44^+^CD69^+^CD103^−^; *p ≤ 0.05 and CD4^+^CD44^+^CD69^+^CD103^+^; *p* = 0.0601) (Figure 3H–J). This group also elicited significant CD8^+^CD44^+^CD69^+^CD103^−^ and CD8^+^CD44^+^CD69^+^CD103^+^ T cell populations (Figure 3K,L). The PUUC+CpG PAL‐NP vaccine formulation also induced a significant and higher CD3^+^ T_RM_ cell population (Figure S2A,B, Supporting Information). PUUC+CpG PAL‐NP formulation elicited a double negative CD4^−^CD8^−^ population in the T cell subset, which could be γδ T cells, probably activated in an MHC‐independent manner and found in epithelial and mucosal tissues (Figure S3J, Supporting Information).

Anti‐viral B cell responses in the local tissues also contribute to long‐term immune protection against SARS‐CoV‐2 and clearing mucosal pathogens.^[^ ^36^ ^]^ Mice immunized with PUUC+CpG PAL‐NP vaccine formulation through IM‐Prime/IN‐Boost route showed a significant and higher RBD tetramer^+^ B cell population (two‐fold), which is specific for receptor binding domain (RBD) of spike protein (Figure 3Q,R). The same vaccine formulation also induced other B cell populations, including antibody‐secreting cells (ASC: B220^+/−^CD138^+^), IgG^+^ antibody‐secreting cells (IgG^+^ASC: B220^+/−^CD138^+^IgA^−^), IgG^+^ resident memory B cells (IgG^+^ B_RM_: B220^+^IgD^−^IgM^−^CD38^+^IgA^−^) and IgM^+^ memory B cells (B220^+^IgM^+^IgD^−^CD38^+^) (Figure S4A,C,E,G, Supporting Information), although these results were not statistically significant. The R848+CpG PAL‐NP induced in IgA^+^ASC (IgA^+^ASC: B220^+/−^CD138^+^IgA^+^) and IgA^+^B_RM_ (B220^+^IgD^−^IgM^−^CD38^+^IgA^+^) cell populations and R848 PAL‐NP group induced GC‐B cells; however, the differences were not statistically significant (Figure S4B,D,F, Supporting Information). The gating strategy for lung B cells is shown in Figure S8B (Supporting Information).

### 2.4 PUUC+CpG and PUUC PAL‐NP Subunit Vaccine Formulation Enhances T_H_1‐Type Cellular Immunity when Delivered IM‐Prime/IN‐Boost

To investigate the T_H_1/T_H_2 expression profile of the T cell population, the restimulated lung cells from spike peptide pool were stained with intracellular T_H_1‐type cytokines (Tumor necrosis factor‐alpha: TNF‐α, Interferon‐gamma: IFN‐γ) and Granzyme B (GrzB), and analyzed with flow cytometry (Figure 3A). PUUC+CpG PAL‐NP formulation exhibited a significant increase in the percentage of monofunctional TNF‐α enriched CD4^+^ and CD8^+^ T_RM_ cell populations (Figure 3M,N). However, PUUC+CpG PAL‐NP formulation significantly increased the frequency of TNF‐α enriched CD4^+^CD44^+^ T_RM_ and GrzB enriched CD8^+^CD44^+^ T cell populations (Figure 3O; Figure S3H, Supporting Information), but did not increase the IFN‐γ enriched CD8^+^ T_RM_ significantly (Figure S2O, Supporting Information). With PUUC alone, there was also an increase in the percentage of IFN‐γ enriched monofunctional CD4^+^ T_RM_ (Figure 3P), although this increase did not reach statistical significance. The PUUC PAL‐NPs significantly increased GrzB expressing monofunctional CD4^+^ T_RM_ and CD4^+^CD44^+^ T cell populations (Figures S2L and S3D, Supporting Information), but failed to increase the significant GrzB expressing CD4^+^CD44^+^ T_RM_ (*p* = 0.0564) cell population (Figure S3I, Supporting Information). None of the adjuvanted PAL‐NP formulations induced significant GrzB expressing CD8^+^ T_RM_ cells in the mice lungs (Figure S2N, Supporting Information).

PUUC+CpG PAL‐NP vaccine formulation led to a notable increase in CD3^+^ T_RM_ population that expresses TNF‐α (Figure S2C, Supporting Information). Although not statistically significant, the PUUC PAL‐NP group elicited a CD3^+^ T_RM_ population expressing IFN‐γ and GrzB (Figure S2D,E, Supporting Information). Both PUUC+CpG and R848+CpG PAL‐NP vaccine formulations significantly increased the CD4^+^, CD8^+^, CD4^+^CD44^+^, and CD8^+^CD44^+^ cell populations in lungs that express TNF‐α (Figures S2F,I and S3B,F, Supporting Information). The PUUC+CpG PAL‐NP formulation significantly elevated the population of monofunctional CD8^+^ T cells expressing GrzB (Figure S2J, Supporting Information). The PUUC PAL‐NP formulation increased the GrzB‐expressed monofunctional CD4^+^ cell population, but this increase lacks statistical significance (Figure S2G, Supporting Information). However, none of the PUUC+CpG PAL‐NP formulation generated the IFN‐*γ* expressed CD4^+^, CD8^+^, CD4^+^CD44^+^, and CD8^+^CD44^+^ T cell populations in the mice lungs (Figures S2H,K and S3C,G, Supporting Information). PUUC PAL‐NP formulation generated CD4^+^ T_RM_ polyfunctional cells that co‐express TNF‐α and IFN‐*γ* (Figure S2M, Supporting Information), and PUUC+CpG PAL‐NP formulation elicited CD8^+^ T_RM_ polyfunctional cells that co‐express both TNF‐α and IFN‐*γ* (Figure S2P, Supporting Information), however, the differences were not statistically significant.

For a more comprehensive study of T_H_1/T_H_2 cytokine profile, we performed a multiplexed cytokine assay to assess various cytokine concentrations from supernatants of lung T cells after restimulation. Secreted cytokine profile was more associated with the T_H_1‐type response, where PUUC+CpG PAL‐NP group induced T_H_1‐type cytokine (IFN‐γ and TNF‐α) secretion, which was consistent with prior assessment by flow cytometry data (Figure S3K,L, Supporting Information). The suppressed (IL‐10, IL‐4) (Figure S3M,N, Supporting Information) or very low detection level of T_H_2‐type cytokine (IL‐13) profile were also observed in PUUC+CpG PAL‐NP vaccinated mice (Figure S3O, Supporting Information).

### 2.5 PUUC+CpG PAL‐NPs Subunit Vaccine Formulation Elicits Comparable SARS‐CoV‐2 Mucosal Humoral Immunity with IN‐Prime/IN‐Boost

**Figure 4:**
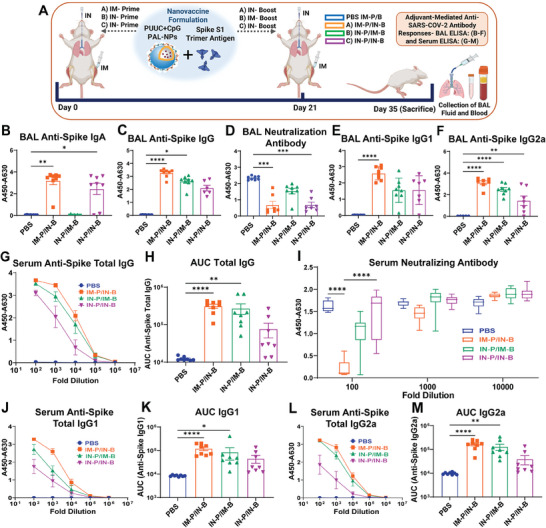
PUUC+CpG dual‐adjuvanted PAL‐NPs protein subunit vaccine formulation elicit robust SARS‐CoV‐2 mucosal and systemic humoral immunity with IM‐Prime/IN‐Boost group and induces a significant level of mucosal humoral responses with IN‐Prime/IN‐Boost group. A) Vaccination study design: Female BALB/c mice (n = 8 for all groups) were immunized using PUUC+CpG PAL‐NP vaccine formulation combined with S1 spike protein (see Table S1, Supporting Information for doses) through three different prime‐boost strategies (day 0: prime and day 21: boost): IM‐Prime/IN‐Boost, IN‐Prime/IM‐Boost, and IN‐Prime/IN‐Boost. On Day 35, BAL fluid and serum samples were collected from vaccinated mice, and assayed using ELISA. B–F) BAL fluid from vaccinated mice was assayed for anti‐spike IgA B), IgG C), spike nAbs D), IgG1 E), and IgG2a F) with ELISA at 1:5 dilution except for IgA and neutralization assay which was performed at 1:2 dilution. G,H) Anti‐spike total IgG in serum at various dilutions measured by absorbance (A450‐A630 nm) and comparison of AUC. I) ACE‐2 signal measured by absorbance (A450‐A630 nm)  in spike protein neutralization assay with ELISA. J,K) Quantification of serum anti‐spike IgG1 at various dilutions and comparison of AUC. L,M) Quantification of serum anti‐spike IgG2a at various dilutions and comparison of AUC. Error bars represent the SEM. Normality was assessed with the Kolmogorov–Smirnov test. Statistical significance was determined with the Kruskal–Wallis test and Dunn's post‐hoc test for multiple comparisons. *p ≤ 0.05, **p ≤ 0.01, ***p ≤ 0.001, ****p ≤ 0.0001 for all graphs.

With PUUC+CpG dual‐adjuvant combination on PAL‐NPs, we observed a strong mucosal humoral and cellular, and systemic humoral responses, when immunized IM‐Prime/IN‐Boost. Thus, we further evaluated this combination in vaccine formulation with different prime‐boost vaccination routes: [A) IM‐Prime/IN‐Boost, B) IN‐Prime/IM‐Boost, and C) IN‐Prime/IN‐Boost], to check whether this combination would modulate the mucosal SARS‐CoV‐2 immunity specific to vaccination routes (**Figure** **4**A). Surprisingly, the IN‐Prime/IN‐Boost also induced substantially and significantly higher BAL IgA levels (1:2 dilution, A450‐A630 = 2.4) (Figure 4B), however, the total IgG levels were comparatively lower than IM‐Prime/IN‐Boost (Figure 4C). Interestingly, a similar level of strong nAbs were induced with both IM‐Prime/IN‐Boost and IN‐Prime/IN‐Boost immunization (at 1:2 dilution) (Figure 4D). Similar to first in vivo study, the IM‐Prime/IN‐Boost group generated more T_H_1‐type immunity (IgG2a‐associated) than IN‐Prime/IN‐Boost and IN‐Prime/IM‐Boost groups (Figure 4E (IgG1) and 4F (IgG2a), and Figure S5A, Supporting Information).

The systemic humoral immune responses (serum) generated through IM‐Prime/IN‐Boost route were consistent with the first in vivo study results, which showed higher and significant total IgG and nAb levels (Figure 4G–I). Interestingly, both IM‐Prime/IN‐Boost and IN‐Prime/IM‐Boost groups showed more T_H_1‐type local humoral immunity, compared to IN‐Prime/IN‐Boost group (Figure 4J,K (IgG1), Figure 4L,M (IgG2a); Figure S5B, Supporting Information). A significant serum IgA level was observed with both IM‐Prime/IN‐Boost and IN‐Prime/IN‐Boost, but it was comparatively lower in the later group (Figure S5C,D).

### 2.6 PUUC+CpG PAL‐NP Subunit Vaccine Elicits Robust SARS‐CoV‐2 T Cell (Tissue‐Resident Memory) Immunity with IN‐Prime/IN‐Boost and B Cell Responses with IM‐Prime/IN‐Boost

**Figure 5:**
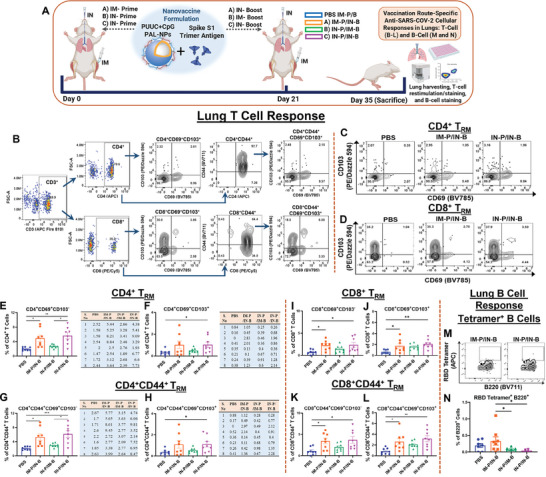
PUUC+CpG dual‐adjuvanted PAL‐NPs protein subunit vaccine formulation elicits robust SARS‐CoV‐2 T cell (T_RM_) immunity with IN‐Prime/IN‐Boost and B cell responses with IM‐Prime/IN‐Boost. A) Vaccination study design: Female BALB/c mice (n = 8 for all groups) were immunized using PUUC+CpG PAL‐NP vaccine formulation combined with S1 spike protein (see Table S1, Supporting Information for doses) through three different prime‐boost strategies (day 0: prime and day 21: boost): IM‐Prime/IN‐Boost, IN‐Prime/IM‐Boost, and IN‐Prime/IN‐Boost. On Day 35, mice were sacrificed, and lung cells were collected and restimulated with spike peptide for 6 h. B) Gating strategies for analysis of CD4^+^ and CD8^+^ T_RM_ responses. C,D) Representative FCM plots of CD4^+^ T_RM_ and CD8^+^ T_RM_ populations in groups: PBS, IM‐Prime/IN‐Boost. E,F) Percentage of CD4^+^CD69^+^CD103^‐^ and CD4^+^CD69^+^CD103^+^ T cells were represented through graphs along with their respective values (n = 8 for all groups) in a table format. G,H) Percentage of CD4^+^CD44^+^CD69^+^CD103^‐^ and CD4^+^CD44^+^ T_RM_ cells were represented through graphs along with their respective values (n = 8 for all groups) in table format. I–L) Percentage of CD8^+^CD44^+^CD69^+^CD103^‐^ I), CD8^+^CD44^+^ T_RM_ J), CD8^+^CD44^+^CD69^+^CD103^‐^ K), and CD8^+^CD44^+^ T_RM_ L) T cell populations. M,N) Representative FCM plots and percentage of RBD tetramer^+^ B220^+^ cells. Error bars represent the SEM. Statistical significance was calculated using one‐way ANOVA followed by Tukey's post‐hoc test for the Figures (E,G, and I–L) and Bonferroni's post‐hoc test for Figures F) and H) for multiple comparisons. *p ≤ 0.05, **p ≤ 0.01, ***p ≤ 0.001, ****p ≤ 0.0001 for all graphs.

We next investigated the vaccination route‐specific cellular immune responses (T cell and B cell) in the lungs after PUUC+CpG PAL‐NPs vaccination in mice (**Figure** **5**A). The gating strategy for lung T cells subset is shown in Figure 5B and Figure S8A (Supporting Information). Surprisingly, PUUC+CpG PAL‐NPs vaccine formulation has induced stronger and enhanced local T cell responses (T_RM_) when delivered IN‐Prime/IN‐Boost, and were also higher than IM‐Prime/IN‐Boost. The frequency of CD4^+^CD69^+^CD103^−^ cells was also higher in IN‐Prime/IN‐Boost group (≈2.73 fold) than IM‐Prime/IN‐Boost group (≈2.28 fold) with respect to the controls (PBS) (Figure 5C,E). Similarly, the frequency of CD4^+^CD69^+^CD103^+^ population was slightly higher in IN‐Prime/IN‐Boost group (≈3.17 fold) than IM‐Prime/IN‐Boost group (≈3.28 fold) (Figure 5C,F).

With CD8^+^ T cell responses, we observed similar results. Mice immunized with PUUC+CpG PAL‐NPs formulation through IN‐Prime/IN‐Boost, showed a higher frequency of CD8^+^CD69^+^CD103^−^ T cells (≈3.3 fold) and were even higher than IM‐Prime/IN‐Boost (≈3.17 fold) (Figure 5D,I). A higher frequency of CD8^+^ T_RM_ population was also observed in mice immunized IN‐Prime/IN‐Boost (≈3.23 fold) than IM‐Prime/IN‐Boost group (≈2.72 fold) (Figure 5D,J). We did not observe significant differences in CD4^+^CD44^+^ and CD8^+^CD44^+^ T cell populations with any vaccination groups (Figures S5O and S6K, Supporting Information). Detailed data of all T cell subsets starting from CD3 to CD4, CD44, CD69, and CD103, along with the comparative gating strategy of CD4^+^ T_RM_ and CD4^+^CD44^+^ T_RM_ in IM‐Prime/IN‐Boost and IN‐Prime/IN‐Boost groups (n = 8) is shown in Supporting Information Figures S5E–G and S5K–R (Supporting Information). The IN‐Prime/IN‐Boost group also showed a significant and higher frequency of other T cell populations: CD4^+^CD44^+^CD69^+^CD103^−^, CD8^+^CD44^+^CD69^+^CD103 and CD8^+^CD44^+^CD69^+^CD103^+^ (Figure 5G,K,L), however, the frequency of CD4^+^CD44^+^CD69^+^CD103^+^ T cells was almost similar as observed with IM‐Prime/IN‐Boost (Figure 5H). We also evaluated the CD3^+^CD4^−^CD8 TCRγδ^+^ (*p* = 0.0507) cell populations which were higher in mice immunized IN‐Prime/IN‐Boost (Figure S6Q, Supporting Information). We observed a significantly higher RBD tetramer^+^ B cell population with IM‐Prime/IN Boost, compared to IN‐Prime/IM‐Boost and IN‐Prime/IN‐Boost groups (Figure 5M,N). The IN‐Prime/IN‐Boost group induced a significant B220^+^ cell population (Figure S7A, Supporting Information), but this group did not significantly induce other B cell populations (IgA^+^ B_RM_, GC‐B cells, and IgM^+^ B_RM_) (Figure S7C–E, Supporting Information). The IM‐Prime/IN‐Boost group induced IgA^+^ ASC population, though the differences were not significant (Figure S7B, Supporting Information).

### 2.7 PUUC+CpG PAL‐NPs Subunit Vaccine Formulation Enhances T_H_1‐Type Immunity with IN‐Prime/IN‐Boost Group

**Figure 6:**
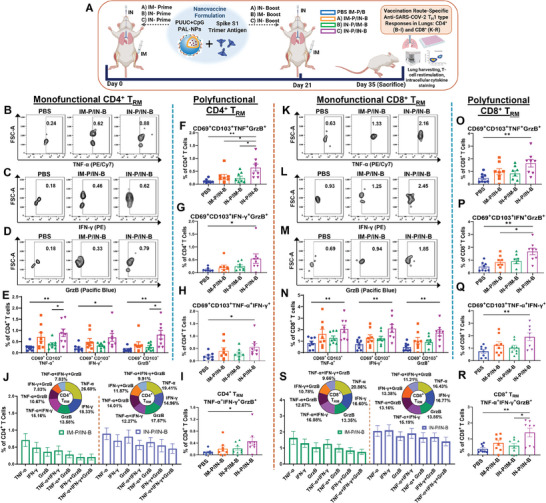
PUUC+CpG dual‐adjuvanted PAL‐NPs subunit vaccine formulation enhances T_H_1‐type immunity with IN‐Prime/IN‐Boost group. A) Female BALB/c mice (n = 8 for all groups) were immunized using PUUC+CpG PAL‐NP vaccine formulation combined with S1 spike protein (see Table S1, Supporting Information for doses) through three different prime‐boost strategies (day 0: prime and day 21: boost): IM‐Prime/IN‐Boost, IN‐Prime/IM‐Boost, and IN‐Prime/IN‐Boost. Mice were sacrificed on Day 35, and the collected lung cells were restimulated with overlapping spike peptides for 6 h and analyzed using flow cytometry to detect antigen‐specific T cell cytokine production. B–D) Representative FCM plots (groups: PBS, IM‐Prime/IN‐Boost, and IN‐Prime/IN‐Boost) of monofunctional CD4^+^ T_RM_ cells expressing TNF‐α B), IFN‐γ C), and GrzB D). E) Percentages of monofunctional CD4^+^ T_RM_ cells expressing TNF‐α, IFN‐γ, and GrzB. F–I) Percentages of polyfunctional CD4^+^ T_RM_ cells expressing TNF‐α+GrzB F), IFN‐γ+GrzB G), TNF‐α+GrzB H), and TNF‐α+IFN‐γ+GrzB I). J) Comparative summary (bar graph) of a percentage of monofunctional and polyfunctional CD4^+^ T_RM_ cells expressing TNF‐α, IFN‐γ, and GrzB in IM‐Prime/IN‐Boost and IN‐Prime/IN‐Boost (donut graph showing the percentage of each group as a fraction of total response). K–M) Representative FCM plots (groups: PBS, IM‐Prime/IN‐Boost, and IN‐Prime/IN‐Boost) of monofunctional CD8^+^ T_RM_ cells expressing TNF‐α K), IFN‐γ L), and GrzB M). N) Percentages of monofunctional CD8^+^ T_RM_ cells expressing TNF‐α, IFN‐γ, and GrzB. O–R) Percentages of polyfunctional CD8^+^ T_RM_ cells expressing TNF‐α+GrzB O), IFN‐γ+GrzB P), TNF‐α+GrzB Q), and TNF‐α+IFN‐γ+GrzB R). S) Comparative summary (bar graph) of percentage of monofunctional and polyfunctional CD8^+^ T_RM_ cells expressing TNF‐α, IFN‐γ, and GrzB in IM‐Prime/IN‐Boost and IN‐Prime/IN‐Boost (donut graph showing the percentage of each group as a fraction of total response). Error bars represent the SEM. Statistical significance was calculated using One‐Way ANOVA and Tukey post‐hoc test for multiple comparisons. *p ≤ 0.05, **p ≤ 0.01, ***p ≤ 0.001, ****p ≤ 0.0001 for all graphs.

We further examined the immunization route‐specific T_H_1 and T_H_2 type immune responses (**Figure** **6**A). We observed a significant and higher monofunctional CD4^+^ T_RM_ and CD8^+^ T_RM_ population that expresses T_H_1‐type intracellular cytokines: TNF‐α, IFN‐γ and cytotoxic Granzyme B (GrzB), with IN‐Prime/IN‐Boost, compared to IM‐Prime/IN‐Boost and IN‐Prime/IM‐Boost groups (Figure 6B–D, (CD4^+^ T_RM_ FCM plots) and Figure 6K–M, (CD8^+^ T_RM_ FCM plots), and Figure 6E,N (percentage)). We also observed significantly higher monofunctional CD3^+^ T_RM_, CD4^+^, CD4^+^CD44^+^, CD8^+^, and CD8^+^CD44^+^ T cell populations enriched for GrzB, with IN‐Prime/IN‐Boost group (Figures S5J and S6A,D,H,L, Supporting Information). The CD3^+^ T_RM_ also expressed a significant TNF‐α but did not express significant IFN‐γ (Figure S5H,I, Supporting Information). The CD4^+^, CD4^+^CD44^+^, CD8^+^, and CD8^+^CD44^+^ T cell populations also did not express significant IFN‐γ and TNF‐α (Figures S6B,C,E,F,I,J and S6M,N, Supporting Information). At the same time, the IN‐Prime/IN‐Boost group showed a significant increase in the frequency of monofunctional CD4^+^CD44^+^ T_RM_ and CD8^+^CD44^+^ T_RM_ cell population that expresses TNF‐α, IFN‐γ, and cytotoxic GrzB, compared to IM‐Prime/IN‐Boost groups (Figure S6O,P, Supporting Information). We also observed the significant and higher polyfunctional CD4^+^ T_RM_ and CD8^+^ T_RM_ cell population, which co‐express TNF‐α and GrzB (Figure 6F,O), IFN‐γ and GrzB (Figure 6G,P), and TNF‐α and IFN‐γ (Figure 6H,Q), with IN‐Prime/IN‐Boost group. Along with that, the IN‐Prime/IN‐Boost group also induced higher polyfunctional CD4^+^ T_RM_ and CD8^+^ T_RM_ cell population, which co‐expresses all three TNF‐α, IFN‐γ, and GrzB (Figure 6I,R). The total fraction of this polyfunctional CD4^+^ T_RM_ and CD8^+^ T_RM_ cell populations were also higher in IN‐Prime/IN‐Boost (CD4^+^ T_RM_: 9.91 and CD8^+^ T_RM_: 11.21) group compared to IM‐Prime/IN‐ Boost (CD4^+^ T_RM_: 7.83 and CD8^+^ T_RM_: 9.66) (Figure 6J,S). However, the secreted cytokine profile was more associated with T_H_1‐type response with both IM‐Prime/IN‐Boost, and IN‐Prime/IM‐Boost groups, which secretes IFN‐γ (Figure S7G, Supporting Information). A very low level (or below the detection limit) of T_H_2 cytokines (IL‐4, IL‐13, IL‐10) were observed with all the groups (Figure S7H–J, Supporting Information).

## 3 Discussion

Vaccines that target the mucosal tissues and induce robust and balanced mucosal‐systemic immunity are required to reduce viral shedding, prevent initial infection, and provide overall protection against SARS‐CoV‐2.^[^ ^10^, ^11^, ^12^, ^13^, ^14^, ^15^ ^]^ Protein subunit nanovaccines formulated with an appropriate combination of adjuvants can represent a promising strategy for developing mucosal vaccines.^[^ ^20^, ^21^, ^29^, ^30^, ^37^, ^38^ ^]^ Moreover, other specific factors such as biopolymer properties, nanovaccine design, prime‐boost immunization routes, vaccine doses, and booster time intervals also significantly modulate SARS‐CoV‐2 immune responses.^[^ ^38^, ^39^, ^40^, ^41^, ^42^ ^]^ Therefore, in this study, we focussed on three important aspects to develop a potent SARS‐CoV‐2 mucosal subunit nanovaccine: a) designing the nanoparticle by changing biopolymer chemistry, which helps to enhance combination adjuvant delivery and immunomodulation, b) screening multiple adjuvant combinations of RLRs and TLRs agonists on nanoparticles for enhanced SARS‐CoV‐2 mucosal immune responses, and c) a nanovaccine administration route‐specific (different prime and boost) and comparative study using screened combination adjuvants NPs.

Cationic polysaccharide biomaterials have excellent mucoadhesive properties and enhance permeability by disrupting tight junctions between epithelial cells.^[^ ^42^, ^43^, ^44^, ^45^ ^]^ However, they exhibit systemic toxicity, due to a high number of primary amines, which can be reduced by modifying them with higher‐order amines.^[^ ^46^ ^]^ We performed chemical/structural modification in polysaccharide polymer to synthesize a polysaccharide‐amino acid‐lipid nanoparticle (PAL‐NPs). Each chemical modification in polysaccharide plays a specific role: a) stearyl lipid core provides nanoparticle stability and enhances encapsulation of hydrophobic adjuvants,^[^ ^47^ ^]^ b) cationic amino acids (arginine/histidine) aids in surface loading of nucleic acid adjuvants and enhanced their delivery,^[^ ^48^, ^49^ ^]^ c) incorporation of disulfide bond maintains the PAL‐NP degradability and enhances the adjuvant's delivery properties.^[^ ^50^ ^]^ The modified polysaccharide‐amino acid polymer demonstrated a strong buffering capacity within the pH range of lysosomal compartments, which can significantly influence and enhance the release of nanoparticulate systems from intracellular endosomal escape pathways.^[^ ^48^, ^49^, ^51^ ^]^ Further, the PAL‐NPs maintained their stability by retaining physiochemical properties (size, zeta potential, and encapsulated adjuvants) for up to a month and can be lyophilized for long‐term storage and use, which also enhances their efficacy to be used for novel subunit nanovaccine. We hypothesized that this improved rational design of adjuvanted PAL‐NPs will aid in achieving a balanced dual‐adjuvant delivery, demonstrating minimal toxicity both in vitro and in vivo and ultimately enhancing vaccine effectiveness.

Initial in‐vitro investigations indicated that adjuvanted PAL‐NPs targeting RLRs (RIG‐I) and TLRs (TLR‐7/8 and TLR‐9) elicit more diverse proinflammatory cytokine responses (including IL12p70, IL‐1β, and IFN‐β) with GM‐CSF differentiated BMDC, likely due to a broader and heterogenous population of APCs (monocytes, monocyte‐derived macrophages, monocyte‐derived DCs, cDCs, and neutrophils), consistent with our previous results.^[^ ^28^, ^32^, ^52^ ^]^ In comparison to PUUC, CpG induces more proinflammatory cytokines (IL‐12p70) and type‐1 interferon (IFN‐β) by activating TLR‐9 early in endosomes, overcoming RIG‐I signaling in the PUUC+CpG combination, thus required higher PUUC doses. This necessitates further in‐vitro optimization of PUUC doses. Cationic PLPs (pathogen‐like particles) typically enhance inflammasome complex activation.^[^ ^53^ ^]^ PUUC PAL‐NPs showed a lower IL‐1β response than PAL‐NPs alone, likely due to the reduced zeta potential from anionic PUUC loading and enhanced NF‐κB activation.^[^ ^54^ ^]^

In vitro investigations of adjuvanted PAL‐NPs (PUUC, CpG, R848, PUUC+CpG, R848+CpG) targeting RLRs and TLRs showed initial innate immune response stimulation. However, due to differences in nanoparticle internalization, complement responses, and cell feedback mechanisms, these findings often do not translate well in vivo.^[^ ^46^ ^]^ Due to the well‐established and previously described limitations of soluble adjuvants, which render them clinically irrelevant, particularly for multi‐adjuvant delivery, we intentionally chose not to include any soluble agonists (PUUC, CpG, and R848) in our in vitro and in vivo study.^[^ ^55^, ^56^ ^]^ Further, in vivo studies of adjuvanted PAL‐NPs (PUUC, R848, PUUC+CpG, and R848+CpG) were needed to assess their efficacy, safety, and potential for future adjuvanted subunit vaccine development. However, we excluded CpG PAL‐NP alone group for in vivo screening, despite its similar in vitro immune response to the PUUC+CpG group, because this combination generated more broad and synergistic behavior in vivo in our earlier reports.^[^ ^30^ ^]^ Also, the higher doses of CpG alone are unsuitable for IN vaccination, so we hypothesized that adding PUUC to reduce the CpG dose can form a better combination adjuvant nanovaccine formulation in vivo.^[^ ^18^ ^]^ A more in‐depth investigation is required to characterize the differential cytokine responses and their specific pathways for distinct immune cell subsets when activated by adjuvanted PAL‐NPs, which is beyond the scope of our current studies.

**Figure 7:**
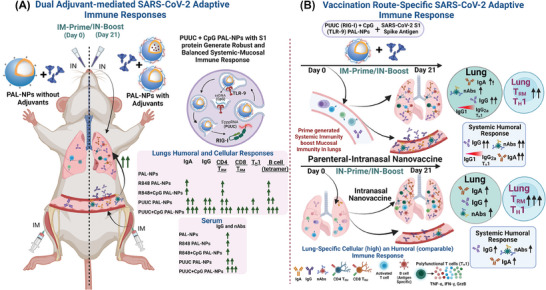
Summary of the adaptive immune responses, including lung‐specific (both humoral and cellular) and systemic (humoral) immune responses, generated in mice following treatment with adjuvanted PAL‐NP nanovaccine formulations and evaluated through two in vivo SARS‐CoV‐2 vaccination studies. A) Investigation of adjuvant‐mediated adaptive immune responses with various adjuvant combinations on PAL‐NPs, delivered with S1 spike trimer antigen via IM‐prime and IN‐boost strategy. Identification of best combination adjuvant PAL nanovaccine that generates robust and balanced mucosal‐systemic responses, thereby enhancing existing IM immunity and triggering potent mucosal SARS‐CoV‐2 immunity. B) Comparison study of vaccination route‐specific immune responses generated in mice after administration of PUUC+CpG PAL nanovaccine through different prime‐boost routes (IM‐P/IN‐B and IN‐P/IN‐B). The purely intranasal route (IN‐P/IN‐B) induced higher T cell responses with T_H_1 type immunity and a comparable humoral response, suggesting its potential use for mucosal vaccine delivery.

Herein, we designed a two‐step in vivo SARS‐CoV‐2 vaccination study using adjuvanted PAL‐NP nanovaccine in a mouse model (**Figure** **7**A,B). The first vivo study helps to choose the best combination adjuvant PAL nanovaccine candidate, which strengthens the existing IM immunity and triggers the potent mucosal SARS‐CoV‐2 immunity (Figure 7A). In the second study, we conducted a comprehensive and comparative study of immunization route‐specific (different prime‐boost) immune responses using the best combination adjuvant nanovaccine candidate (Figure 7B). For in vivo studies, we have used the pre‐optimized doses of the adjuvants,^[^ ^28^, ^29^, ^30^, ^56^ ^]^ antigens,^[^ ^30^ ^]^ and PAL‐NPs for both intramuscular (IM) and intranasal (IN) vaccinations to ensure that PUUC+CpG PAL nanovaccine demonstrates immune‐enhancing effect within the safety parameters.^[^ ^2^, ^57^ ^]^ The first in vivo study revealed that PUUC+CpG PAL‐NPs is the best combination (dual) adjuvanted nanovaccine, which induces high‐quality and robust mucosal humoral (IgA and IgG), systemic humoral (IgG and nAb), and local cellular immune responses (heavily favoring T_H_1 responses), when delivered IM‐Prime/IN‐Boost. This makes PUUC+CpG PAL‐NPs formulation more suitable for a mucosal subunit vaccine. Mucosal IgA is the major antibody response that restricts the entry of respiratory viral pathogens. It is the most dominating antibody during early immune response after infection and is also responsible for sterilizing immunity.^[^ ^10^, ^58^ ^]^ The PUUC+CpG PAL‐NPs group induced T_H_1 polarized antibody response (IgG2a switching) in both BAL fluid and serum, which are also observed in the previous antiviral RIG‐I agonists‐based influenza vaccines research study.^[^ ^59^ ^]^ Thus, our findings highlight the importance of RIG‐I agonists in fighting respiratory viral diseases.^[^ ^59^ ^]^ Despite being a preclinical study on mice, this result can be correlated to human humoral responses, especially the human IgG1, which is analogous to mice IgG2a isotypes and is the most preferred subclass of IgG antibody that exhibits optimum antiviral activity.^[^ ^60^ ^]^ IN‐boosted, PUUC+CpG PAL‐NPs after IM priming are the potent inducer of lung‐specific T_RM_ cell responses (CD4^+^, CD8^+^, and CD4^+^CD44^+^) compared to other adjuvant combinations except for PUUC PAL‐NPs, which also induces a significant CD4^+^ T_RM_ population. The CD8^+^ tissue‐resident memory T cells (T_RM_) are known to be more effective for viral clearance, and CD4^+^ T_RM_ is involved in a broad spectrum of activities, including the durability of nAbs responses and promoting the development of protective CD8^+^ T_RM_ and memory B cells.^[^ ^14^, ^35^, ^61^, ^62^ ^]^ Balancing mucoadhesiveness and mucus permeation is important for nanovaccine delivery in the respiratory tract. Mucoadhesiveness aids retention, while permeation enhances lung delivery.^[^ ^42^, ^43^, ^44^, ^45^ ^]^ Although upper respiratory tract responses weren't assessed, BAL fluid from the trachea and lungs showed strong humoral responses. Similarly, the local cellular responses were also high, which confirms that modified polysaccharide nanoparticles balance both mucus adhesion and permeation, establishing an innovative mucosal nanovaccine system.

Previous studies on alum adjuvanted RSV vaccines and few preclinical SARS‐CoV research have shown that vaccine‐associated enhanced respiratory disease (VAERD), has a connection with CD4 T_H_2‐type response.^[^ ^63^, ^64^ ^]^ However, our results showed that the RIG‐I targeted PUUC+CpG and PUUC PAL‐NP group induces T_RM_ in the lungs_,_ which expresses T_H_1‐type cytokines with suppressed or very low detection levels of T_H_2‐type cytokines. Therefore, both the PUUC+CpG and PUUC PAL‐NPs groups, which share a RIG‐I agonist as a common adjuvant, significantly enhanced the magnitude of T_RM_ responses, polarized it to T_H_1 profile, and led to potent antiviral immunity without showing pathogenic T_H_2‐type responses. Induction of antigen‐specific RBD tetramer^+^ B cells with PUUC+CpG PAL‐NP group signifies the antigen encounter and further B cell activation and formation of memory B cells.^[^ ^36^ ^]^

Although adjuvants can play a crucial role in enhancing potent antiviral mucosal immunity, most studies investigating their effectiveness have focused on IM vaccines with limited knowledge about the role in mucosal vaccines.^[^ ^21^, ^22^, ^37^ ^]^ For example, in humans, CpG (TLR‐9 agonist) based subunit vaccines elicit a systemic immune response when administered IM and are not an ideal adjuvant candidate for IN immunization.^[^ ^65^ ^]^ Few recent studies focused on RIG‐I and TLRs targeted SARS‐CoV‐2 protein‐subunit vaccines can provide a useful comparison point for our study.^[^ ^23^, ^24^, ^25^ ^]^ Nguyen et al. developed a C.S./CpG/RBD intranasal vaccine that induces mucosal humoral response and has demonstrated efficacy against VOCs but lacks to generate local T cell immunity.^[^ ^24^ ^]^ Jangra et al. presented a preclinical study showing that a three‐dose nanoemulsion RIG‐I agonist (IVT DI) adjuvanted SARS‐CoV‐2 subunit IN vaccine results in systemic T_H_1 and only IgG responses in both BAL fluid and serum, but did not observe an enhanced lung‐specific humoral and memory T cell response.^[^ ^23^ ^]^ The aim of using mucosal vaccines is to establish protective local antiviral immunity. While preclinical studies have shown some success in achieving this, they have not fully met the criteria for overall protection. Therefore, it is essential to advance the current SARS‐CoV‐2 vaccine strategies by incorporating combination adjuvants. In contrast, a recent preclinical study by Tianyang et al. showed that IM priming of m‐RNA LNP and IN‐Boost spike protein only (unadjuvanted) elicits protective SARS‐CoV‐2 immunity.^[^ ^16^ ^]^ In our study, we studied how adjuvants, specifically combination adjuvants induce or improve mucosal and systemic immunity. We showed that the dual‐adjuvanted PUUC+CpG PAL‐NP group offers an elevated level of mucosal antiviral immunity, both humoral and cellular, as well as a systemic humoral immune response with the IM‐Prime/IN‐Boost strategy. Also, robust and broad local T cells and comparable mucosal humoral (IgA and nAb) immune responses were induced with IN‐Prime/IN‐Boost. RIG‐I is triggered by the native SARS‐CoV‐2 virus, so we hypothesized that it can mimic infection. In comparison to the unadjuvanted approach, our results have revealed significant benefits of using adjuvants.

Different vaccine administration routes have different mechanisms to induce an immune response.^[^ ^40^ ^]^ IM‐Prime and IN‐Boosted PUUC+CpG dual‐adjuvant nanovaccine showed consistent results with both the in vivo studies, which can also be compared with recent preclinical SARS‐CoV‐2 mucosal vaccine studies, where IM‐prime generated immunity is significantly enhanced by IN‐Boosting.^[^ ^16^, ^18^ ^]^ Interestingly, IN‐Prime/IN‐Boost group generated the high level of lung T cell immunity along with good local humoral responses (IgA and nAb), compared to IM‐Prime/IN‐Boost group. Along with a strong T cell response and a comparable mucosal humoral response, the IN‐Prime/IN‐Boost group showed a considerable systemic IgG response but a relatively lower systemic nAb response than the other two groups, which include IM vaccination in either the prime or boost. The IN‐Prime/IN‐Boost group induced robust monofunctional and polyfunctional T_RM_ cells (CD4^+^ and CD8^+^) that express T_H_1‐type intracellular cytokines: TNF‐α and IFN‐γ, and GrzB, but not the pathogenic T_H_2‐type. The large population of polyfunctional T cells producing multiple T_H_1 cytokines demonstrates the potential for adequate antigen‐presentation and strong co‐stimulation from professional APCs.^[^ ^61^ ^]^ A similar trend of T cell and cytokine data was observed in the study on recovered SARS‐CoV‐2 patients by Grifoni et al., which showed that T cell responses appeared as T_H_1 phenotype with lower levels of T_H_2‐type response.^[^ ^61^, ^66^ ^]^ As SARS‐CoV‐2 continuously evolves with new immune evasive variants, the population needs to be boosted with the new‐generation potent mucosal vaccines that provide enhanced protection and reduced transmission while maintaining their safety and efficacy. Although our results focused on only nanoparticle‐based subunit vaccines, if used as a booster, we believe this vaccine design would broadly apply to other primary immunization methods.

## 4 Conclusion

This study presents the development of a newly designed combination adjuvanted (PUUC RNA: RIG‐I agonist and CpG DNA: TLR‐9 agonist) subunit SARS‐COV‐2 nanovaccine and its novel approach of immunization (parenteral prime and intranasal boost), which effectively induced a potent and balanced mucosal‐systemic antiviral immunity. Promising outcomes from the intranasal prime and boost PUUC+CpG nanovaccine delivery also suggest the possibility of a fully mucosal delivery route. Our preclinical study demonstrates that the mucosal (IN) route is essential for new vaccination strategies and should be included in the current immunization protocols. Future research would assess the potential of PUUC+CpG adjuvant combination on PAL‐NPs against direct challenge with SARS‐CoV‐2 variants. Additionally, the effectiveness of this vaccine in the upper respiratory tract (nasal mucosal immunity) could be evaluated in future studies. Our results are highly promising in preclinical studies, but due to the inherent immunological differences between animal models and humans, it requires further optimization for future clinical and translational use.

## 5 Experimental Section

All animal experiments were conducted in accordance with approved IACUC (Institutional Animal Care and Use Committee) protocols (reference number #A100207) by the Georgia Institute of Technology.

### Synthesis of PAL‐NPs and Adjuvant Loading

Amphiphilic polysaccharide‐amino acid‐lipid (PAL) polymer and cationic polysaccharide‐amino acid‐lipid nanoparticles (PAL‐NPs) were synthesized and characterized as described in the Supporting Information. Synthesis of adjuvanted PAL‐NPs (loading of multiple and combination adjuvants) and their characterization are described in the Supporting Information (see Figure S1A, Supporting Information).

### In Vitro Activation of BMDCs

Detailed methods of BMDC culture and activation using PAL‐NPs are described in Supporting Information.

### Adjuvanted PAL Subunit Nanovaccine Formulation

Final nanovaccine formulations were prepared by mixing the adjuvanted PAL‐NPs (250 µg per mice) and recombinant, stabilized SARS‐CoV‐2 spike S1 trimer protein (1 µg per mice, SPN‐C52H9, ACRO Biosystems) in PBS (pH = ≈7.4). The doses of adjuvants on the PAL‐NP formulation for the IM vaccination (per mice) were 20 µg for PUUC and R848, and 40 µg for CpG, and also shown in Table S1 (Supporting Information). For IN vaccination, all doses were the same except the CpG, which was reduced to 20 µg.

### In Vivo Vaccination Studies and Doses

BALB/c female mice (6–8 weeks old Jackson Labs, Bar Harbor, ME) were used to measure the adaptive immune response and anesthetized using 30% v/v Isoflurane diluted in propylene glycol for vaccination. For IM vaccination, formulations were prepared in a total of 100 µL of PBS (10 mm), out of which 50 µL was injected in both the right and left anterior tibialis muscle. For IN vaccination, the formulations were prepared in 40 µL of PBS (10 mm), out of which 20 µL was administered dropwise in both the left and right nares.

### Sample Collection, Tissue Processing, Flow Cytometry, and Ex Vivo Cytokine Analysis

On Day 35, the mice were sacrificed, and samples (serum, BAL fluid, and lungs) were collected. Detailed methods for all experiments related to sample collection, purification, tissue processing into single‐cell suspensions, T cell restimulation, T cell and B cell staining, RBD tetramer synthesis, and flow cytometry are described in the Supporting Information.

### Quantification of Anti‐Spike SARS‐CoV‐2 Antibody Responses

Enzyme‐linked immunosorbent assays (ELISAs) were performed to quantify anti‐spike SARS‐CoV‐2 antibody (IgA, total IgG, IgG1, and IgG2a) responses in BAL fluid and serum. The detailed method is described in the Supporting Information.

### Modified ELISA Assay to Measure Anti‐Spike Neutralizing Antibodies

nAbs in both BAL fluid and serum were assessed by measuring the inhibition of the Spike RBD‐ACE2 interaction using an ELISA‐based ACE2 competition assay. For the quantification of nAbs, the above‐described ELISA method for antibody responses was slightly modified, where instead of a secondary antibody, ACE2 protein was used. Lower absorbance values indicated higher spike‐neutralizing antibody levels. The detailed method is described in the Supporting Information.

### Statistical Analysis and Graphics Preparation

All flow cytometry FCS files were analyzed with FlowJo (v10, B.D.). Statistical analyses were performed with GraphPad Prism 9. For comparisons of more than two groups, statistical differences between normally distributed datasets were determined with a one‐way analysis of variance (ANOVA) followed by Tukey's and Bonferroni's post‐hoc test for multiple comparisons. Similarly, nonparametric datasets were evaluated with the Kruskal–Wallis test and Dunn's post‐hoc test. *p* ≤ 0.05 was considered statistically significant. Structural representations of polymers were drawn in ChemDraw software. Graphpad plots were arranged in Adobe Illustrator, and vaccination figures were made with Biorender.

## Conflict of Interest

The authors declare no conflict of interest.

## Author Contributions

A.J., E.B., R.J., A.B., D.M., J.H., L.F., and C.V. contributed equally to this work. B.P. and K.R. conceptualized and designed the study. B.P. and K.R. developed the methodology. B.P., Z.W., A.J., E.B., R.J., A.B., D.M., J.H., C.V., L.O.F., D.J.H., R.K.N., R.R., C.S., and M.A.O. conducted the investigation. B.P. and Z.W. created the visualization. B.P. and K.R. supervised the work. B.P. and K.R. wrote the original draft. B.P. and K.R.revised and edited the manuscript.

## Supporting information



Supporting Information

## Data Availability

The data that support the findings of this study are available in the supplementary material of this article.
