# Supplementary material for: A Dual‐Adjuvanted Parenteral‐Intranasal Subunit Nanovaccine generates Robust Systemic and Mucosal Immunity Against SARS‐CoV‐2 in Mice
Source: Adv Sci (Weinh). 2024 Oct 1;11(45):2402792. doi: 10.1002/advs.202402792 (PMC11615772; doi:10.1002/advs.202402792)
Supplement: Supplementary file 1 — Supporting Information [file ADVS-11-2402792-s001.docx]

**Supplementary Information for**

**A Dual-Adjuvanted Parenteral-Intranasal Subunit Nanovaccine generates Robust Systemic and Mucosal Immunity against SARS-CoV-2 in Mice**

***Authors:*** *Bhawana Pandey, Zhengying Wang, Angela Jimenez,^†^ Eshant Bhatia,^†^ Ritika Jain,^‡^ Alexander Beach,^‡^ Drishti Maniar,^§^ Justin Hosten,^§^ Laura O’Farrell,^||^ Casey Vantucci,^||^ David Hur, Richard Noel, Rachel Ringquist, Clinton Smith, Miguel A. Ochoa, Krishnendu Roy^*^*

B. Pandey, Z. Wang, A. Jimenez, R. Jain, D. A. Beach, Maniar, J. Hosten, C. Vantucci, D. Hur, C. Smith, M. A. Ochoa

Wallace H. Coulter Department of Biomedical Engineering, Georgia Institute of Technology, Atlanta, Georgia, USA

E. Bhatia

Woodruff School of Mechanical Engineering, Georgia Institute of Technology, Atlanta, GA, USA

R. Ringquist

The Parker H. Petit Institute for Bioengineering and Biosciences, School of Chemical & Biomolecular Engineering, Georgia Institute of Technology, Atlanta, Georgia, USA

L. O’Farrel and R. Noel

Physiological Research Laboratory, Georgia Institute of Technology, Atlanta, Georgia, USA.

Krishnendu Roy

Wallace H. Coulter Department of Biomedical Engineering, The Parker H. Petit Institute for Bioengineering and Biosciences, Marcus Center for Therapeutic Cell Characterization and Manufacturing, Georgia Institute of Technology, Atlanta, Georgia, USA

Department of Biomedical Engineering, Department of Chemical and Biomolecular Engineering, Vanderbilt University, Department of Pathology, Microbiology, and Immunology, Vanderbilt University School of Medicine, Nashville, Tennessee, USA

Email Address: [krish.roy@vanderbilt.edu](mailto:krish.roy@vanderbilt.edu)

**^†, ‡, §, ||^These authors contributed equally to this work**

**Keywords:** SARS-CoV-2 subunit nanovaccine, mucosal immunity, parenteral and intranasal vaccination, antiviral immunity, polymer nanoparticles, combination adjuvants

**TABLE OF CONTENTS**

**1. Materials**

**2. Experimental methods section: Synthesis of PAL polymer and adjuvanted PAL-NPs**

**2.1. Synthetic steps for polysaccharide (chitosan)-amino acid-lipid polymer: multistep synthesis**

2.1.1. Synthesis of O-carboxymethyl-chitosan: OCMC

2.1.2. Synthesis of thiolated O-carboxymethyl-chitosan: OCMC-SH

2.1.3. Synthesis of cysteamine conjugated OCMC: OCMC-S-S-Cys

2.1.4. Synthesis of arginine and histidine conjugated OCMC-S-S-Cysteamine: OCMC-S-S-(A/H)

2.1.5. Synthesis of polysaccharide-amino acid-lipid (PAL) polymer: OCMC-S-S-(A/H)-SA

2.1.6. Synthesis of PAL-NPs and multiple adjuvant loading

**2.2. Characterization Methods**

2.2.1. Quantification of free thiol content in polymers by Elmann’s assay

2.2.2. NMR analysis of polymers

2.2.3. Estimation of the buffering capacity

2.2.4. Characterisation of adjuvant loading on PAL-NPs

2.2.5. Nanoparticle’s stability

2.2.6. Transmission electronic microscopy (TEM)

2.2.7. Adjuvant release study from PAL-NPs

**2.3. Immunological studies:**

2.3.1. In vitro activation of mouse BM-APCs with combination adjuvants of PAL-NPs formulations

2.3.2. Euthanasia and sample collection (BAL fluid, blood, and lungs)

2.3.3. Ex vivo lung cell restimulation and T cell staining

2.3.4. B cell staining and flow cytometry

2.3.5. SARS-CoV-2 RBD B-cell tetramer synthesis

2.3.6. Supernatant cytokine profile

2.3.7. ELISA assay for quantifying anti-spike antibody responses

2.3.8. Modified ELISA assay to measure anti-spike neutralizing antibodies

**3. Figures**

**Figure S1**. Synthetic scheme of PAL polymer and PAL-NPs characterization.

**Figure S2.** PUUC+CpG PAL subunit nanovaccine formulation with S1 spike protein, elicits robust SARS-CoV-2 elicits T cell immunity when delivered IM-Prime/IN-Boost

**Figure S3.** PUUC+CpG PAL subunit nanovaccine formulation with S1 spike protein, elicits robust SARS-CoV-2 elicits T cell immunity when delivered IM-Prime/IN-Boost

**Figure S4.** Analysis of lung B cell responses when adjuvanted PAL subunit nanovaccine formulations are delivered to mice via IM-Prime/IN-Boost vaccination

**Figure S5.** PUUC+CpG PAL subunit nanovaccine formulation with S1 spike protein, elicits robust SARS-CoV-2 lung-specific T cell immune response with IN-Prime/IN-Boost strategy

**Figure S6.** PUUC+CpG PAL subunit nanovaccine formulation with S1 spike protein elicits robust SARS-CoV-2 T cell immune responses with IN-Prime/IN-Boost route

**Figure S7.** Lung-specific B cell and T cell (secreted cytokine) responses, when PUUC+CpG PAL subunit nanovaccine formulation and mixed with S1 spike protein, delivered with three different prime-boost routes

**Figure S8.** Gating strategies for analysis of adaptive immune responses in the lungs

**Figure S9:** 400 MHz ^1^H NMR spectrum of the carboxylated chitosan (OCMC) in D_2_O with 1% DCl

**Figure S10:** 400 MHz ^1^H NMR spectrum of the thiolated OCMC in D_2_O with 1% DCl

**Figure S11:** 400 MHz ^1^H NMR spectrum of the OCMC-S-S-Cys in D_2_O with 1% DCl

**Figure S12:** 400 MHz ^1^H NMR spectrum of the OCMC-S-S-(A/H) in DMSO-d^6^

**Figure S13:** 400 MHz ^1^H NMR spectrum of the OCMC-S-S-(A/H)-SA in DMSO-d^6^

**4. Table S1: Adjuvanted PAL-NPs formulations for in vitro and in vivo studies**

**5. References**

**1. Materials**

Chitosan polysaccharide (Mw 15 KDa) was purchased from Polysciences (85% degree of deacetylation). Dialysis tubing (MWCO 3.5 kDa, 10 kDa) was purchased from Thermo-Fisher Scientific. NMR solvents and other solvents for synthesis, such as ethanol and diethyl ether, were purchased from Sigma Aldrich.

**2. Experimental methods section: Synthesis of PAL polymer and adjuvanted PAL-NPs**

**2.1. Synthetic steps for polysaccharide (chitosan)-amino acid-lipid polymer: multistep synthesis**

**2.1.1. Synthesis of O-carboxymethyl-chitosan: OCMC**

O-Carboxymethyl-Chitosan (OCMC) was synthesized by following the previously described procedure to increase the selective O-carboxylation and reduce the N-carboxylation^[1]^. The C-6 position of chitosan polysaccharide (500 mg) was first alkalized with 50% aqueous NaOH (20 mL) at -10 °C for one hour. The alkalized polysaccharide was further reacted with 2.5 g monochloroacetic acid (Sigma Aldrich) at 45-55 °C for 6 h. The reaction mixture was added with 70% ethanol to prepare the sodium salt of OCMC, which was further purified by vacuum filtration. The OCMC sodium salt was washed with 70% ethanol and acidified with 1 N HCl to form OCMC. The obtained OCMC was filtered and dried under a vacuum for further use. The incorporation of O-carboxymethyl group at the C-6 position was confirmed by ^1^H NMR (**Fig. S9**).

**2.1.2. Synthesis of thiolated O-carboxymethyl-chitosan: OCMC-SH**

Thiolated OCMC was synthesized by modifying the previously published procedure^[2]^. Briefly, synthesized OCMC was thiolated by covalent conjugation of carboxyl of thioglycolic acid with the amine group of chitosan (C-2 position) using carbodiimide chemistry. Firstly, the carboxyl group of TGA (500 mg, Sigma Aldrich) groups was activated with 1-ethyl-3-(3-dimethylaminopropyl) carbodiimide hydrochloride (EDC, Thermo Fischer) at pH 6.5 in DI water with a final concentration of 125 mM for 2 h. 250 mg of OCMC was acidified with 1M HCl. OCMC solution was added to the activated TGA solution, and the pH of the reaction medium was adjusted to 5 to avoid the formation of a disulfide bond. To eliminate excess TGA and to purify the thiolated OCMC, the reaction mixtures were dialyzed five times using dialysis membrane 10 kDa MWCO (Sigma Aldrich) for two days in the dark against HCl (5 mM), then two times against HCl (5 mM) with 1% NaCl at 10 °C, which helps quench the ionic interactions between anionic sulfhydryl and the cationic polymer. Final dialysis was performed against 1 mM HCl to maintain the pH of the thiolated OCMC polymer to 4. Polymers were further lyophilized and stored at 4°C until further use. Thiolation was confirmed by using ^1^H NMR (**Fig. S10**) and Elmann’s assay (**Fig. S1B**).

**2.1.3. Synthesis of cysteamine conjugated OCMC: OCMC-S-S-Cys**

The above lyophilized thiolated OCMC was first reduced with DTT (Dithiothreitol, Sigma Aldrich) before cysteamine conjugation. This necessary reduction step reduced the disulfide bond formed during lyophilization and helped in the increment of free sulfhydryl groups. For reduction, the thiolated OCMC-SH solution was prepared in DI water, and pH was maintained at 8 using 1 M NaOH. DTT was added in a final concentration of 100 mM, and the reaction mixture was continuously stirred at RT. After approximately two hours, NaCl was added to the reaction mixture (final concentration 1%, weight/volume), and the pH was adjusted to 4 with 1 M HCl. The resulting solution was dialyzed using a similar method as discussed in step 2.1.2. Finally, the solution was filtered through a 0.5 μm filter. The second cycle with DTT was repeated to improve the reduction of remaining disulfide bonds. The filtered thiolated OCMC was conjugated to cysteamine by forming a disulfide bond between thiols of cysteamine (Sigma Aldrich) and free thiols on OCMC. Cysteamine solution was prepared in 1% acetic acid, and the solution pH was adjusted to 6.0. The thiolated OCMC was added dropwise over a 3 h period using an addition funnel to the cysteamine solution. The reaction mixture was allowed to stir for 24 h at RT, and the pH of the final solution was maintained between 4-5. The resulting polymer conjugates were isolated using a similar dialyzing method, as discussed in step 2.1.2. Polymers were further lyophilized and stored at 4 °C until further use. Synthesis of OCMC-S-S-Cys was confirmed by using ^1^H NMR (**Fig. S11**) and Elmann’s assay (**Fig. S1B**). The concentration of free thiol (-SH) groups was decreased after disulfide formation, as shown by Elmann’s assay, which confirms the synthesis of OCMC-S-S-Cys formation.

**2.1.4. Synthesis of arginine and histidine conjugated OCMC-S-S-Cysteamine: OCMC-S-S-(A/H)**

The coupling of both amino acids: N-Boc Histidine (Alfa Aesar) and N-Boc Arginine (Alfa Aesar), onto cysteamine conjugated OCMC was performed by the reaction of the amine groups of OCMC-S-S-Cys and carboxylic group of amino acid in the presence of coupling agents 1-ethyl-3-(3-dimethylaminopropyl) carbodiimide hydrochloride (EDC, Thermo Fischer) and N-hydroxy Succinimide (NHS, Sigma Aldrich). The Boc-protected amino acids were used to reduce the cross-reaction of the free carboxyl group of the C-6 position of chitosan with the amine groups of amino acids. The free carboxyl group of N-Boc Histidine (0, 5, 10 mM) and N-Boc Arginine (0, 10, 20 mM) was first activated individually by the addition of EDC/NHS (10 molar excess) in TEMED/HCL buffer (1% concentration, v/v) at pH 5.5 (Tetramethylethylenediamine, Sigma Aldrich) for 2 h at 25 °C. The activated amino acid solution was added dropwise to the solution of OCMC-S-S-Cys in the same buffer and reacted for the next 16 h. The concentration of both amino acids was used with different ratios to yield conjugates with different degrees of substitution. The pH of the final reaction mixture was maintained at 6. Then, the reaction product was extensively dialyzed against distilled water for two days, and the pH was maintained at 6.5 to remove the unreacted components. The purified polymer was then recovered by lyophilization and stored at 4 °C. The incorporation of arginine and histidine in the polymer chain was confirmed by ^1^H NMR (**Fig. S12**).

**2.1.5. Synthesis of polysaccharide-amino acid-lipid (PAL) polymer:** **OCMC-S-S-(A/H)-SA**

OCMC-S-S-(A/H)-SA was synthesized by the coupling of the carboxyl group (C-6 position) of OCMC-S-S-(A/H) with the amine group of stearyl amine (TCI Chemicals). In brief, the carboxyl group of OCMC-S-S-(A/H) (250 mg) at the C-6 position was activated by the addition of EDC and NHS in 20 mL PBS (pH 6, 10 mM) for two hours. Different amount of stearyl amine (0.25–0.625 mol/mol glucosamine residues) was used to react with carboxyl groups of OCMC-S-S-(A/H). The stearyl amine was pre-dissolved in 20 mL ethanol by heating at 60 °C in a separate round bottom flask. After two hours, the stearyl amine solution was added dropwise to the OCMC-S-S-(A/H) polymer solution by maintaining a similar temperature at 60 °C and again heated to 80 °C. After 6 h, the reaction mixture was allowed to cool to room temperature and again stirred for 18 h. For purification, the reaction mixture was vigorously dialyzed (MWCO 3.5 KDa) against distilled water for 48 h to remove water-soluble by-products and ethanol. The dialyzed suspension was lyophilized and rinsed several times with hot ethanol and diethyl ether and precipitated in ethanol to remove unreacted stearyl amine. Boc deprotection of the amino acids conjugated at the C-2 position of OCMC-S-S-(A/H)-SA (200 mg) was performed using 2 M HCl in dioxane (2 mL) and trifluoroacetic acid (TFA) in ice-cold temperature under an argon atmosphere and stirred for 15 min and further stirred for next three hours at RT. The reaction product was further precipitated in ethanol, washed, and dried. The residue was dialyzed against 0.01 N HCl by redissolving in DI water using dialysis tubing of 3.5 kDa MWCO. The samples were initially dialyzed against 0.01 N HCl for one day and then with DI water for another day with several water changes. ^1^H NMR confirmed the incorporation of stearyl chains in the OCMC-S-S-(A/H) polymer (**Fig. S13**).

**2.1.6.** **Synthesis of PAL-NPs and multiple adjuvant loading**

Cationic and biodegradable polysaccharide-amino acid-lipid nanoparticles (PAL-NPs) were synthesized by probe sonication of an amphiphilic PAL polymer (final concentration of 0.5 mg mL^-1^). The polymer was first hydrated and dispersed overnight in phosphate buffer saline (PBS, pH 7.2, 10 mM). The hydrated polymer was mixed with DMSO (PBS: DMSO ratio, 80:20), and probe sonicated on ice for 10 min. Nanoparticles were purified with vigorous dialysis in PBS (pH 7.2, 10 mM) for one day by changing water thrice. R848 adjuvant encapsulated cationic PAL-NPs (0.5 µg R848 per mg) were synthesized by adding R848 stock in DMSO, followed by probe sonication and dialysis. Nanoparticles were concentrated according to the volume required for the in vivo and in vitro studies. Nanoparticles were electrostatically loaded with nucleic acid adjuvants, CpG ODN 2395 (Invitrogen, Cat# tlrl-2395) and PUUC [3] in 10 mM sodium phosphate buffer (PBS, pH= ~7.0) and left for rotation for 24 h (See table S1 for adjuvant doses). All adjuvants, antigen stock (except R848), and buffers were prepared in nuclease-free water. PUUC RNA was synthesized and characterized following the previously published procedure (*3*). Characterization of adjuvant loading on nanoparticles was described in section 2.2.3.

**2.2. Characterization Methods**

**2.2.1. Quantification of free thiol content in polymers by Elmann’s assay**

To confirm the synthesis of OCMC-S-S-Cys and reduced concentration of free thiols in OCMC-S-S-Cys polymer compared to OCMC-SH, we performed the Elmann’s assay of OCMC after and before thiolation as well as after disulfide bond formation. In brief, a reaction buffer was prepared using sodium phosphate (0.1 M) and ethylenediaminetetraacetic acid (EDTA, 1 mM) at pH 8.0. The stock solution of Elmann’s reagent was prepared by dissolving 2 mg of 5,5′-dithiobis-(2-nitrobenzoic acid) in 0.5 mL same reaction buffer. As per the manufacturer’s instructions, 500 μL of the sample (1 mg of polymer in 1 mL of reaction buffer) was added to a test tube containing 100 μL Elmann’s reagent and 5 mL of reaction buffer. The samples were incubated for an optimized time at 37 °C and protected from light. A 100 µl of the sample was transferred to a 96-well plate. Samples were analyzed using a microplate reader (BIOTEK Synergy HT plate reader, Gen5 software) at a wavelength of 485 nm to determine the content of thiol groups. For the estimation of disulfide contents, OCMC-S-S-Cys polymers were first reduced with NaBH_4_ and then evaluated by Elmann’s reagent. A serial dilution of cysteine hydrochloride monohydrate was used as a standard, and a standard curve was generated using eight serial concentrations of 1.5, 1.25, 1.0, 0.75, 0.5, 0.25, 0.125, 0.0625, and 0 mM. All experiments were performed in triplicate. The free thiol content was quantified according to the following equation 1:

$$Thiolation \%=\frac{\left( OCMC\cdots SH-OCMC \right)}{\left( OCMC\cdots SH \right)}\times100$$

Where OCMC and OCMC-SH stands for carboxylated chitosan and thiolated carboxylated chitosan, respectively.

**2.2.2. NMR analysis of polymers**

The ^1^H NMR analysis was performed on a Bruker Avance III 400 at 25 °C. OCMC, OCMC-SH, OCMC-S-S-Cys polymers were dissolved in D_2_O with 1% DCl. OCMC-S-S-(A/H) and OCMC-S-S-(A/H)-SA polymers were dissolved in deuterated dimethyl sulfoxide (DMSO-d6). Chemical shifts were recorded in parts per million (ppm) using the signal of TMS as the internal reference. NMR spectral data were analyzed using MestreNova NMR software. The incorporation of functional groups in polysaccharides was confirmed by proton NMR spectroscopy. The characteristic peak at 7.1-7.4 ppm appeared due to a guanidine functional group in arginine, suggesting the successful arginine grafting in polysaccharides. Additionally, a characteristic peak of imidazole ring protons at 8.6 ppm and 7.6 ppm confirmed histidine grafting on the polysaccharide backbone. The peaks at 1.2 ppm (-CH_2_) and 1.6 ppm (-CH_3_) in the ^1^H NMR spectrum of amphiphilic chitosan polymer confirmed the successful incorporation of the stearyl chain in chitosan.

**2.2.3. Estimation of the buffering capacity**

The solution of modified polysaccharide amino acid polymer and chitosan solutions was prepared in sodium acetate buffer (pH 5.5) with a concentration of 10 µM. The solutions were further titrated with 0.1 N NaOH. The pH was recorded using a pH meter, and the buffering capacity was compared.

**2.2.4. Characterisation of adjuvant loading on PAL-NPs**

Nanoparticle size and surface zeta potential before the anionic adjuvant loading were measured with a Zetasizer Nano Z.S. (Malvern), as shown in Table S1. The sample preparation details for TEM were provided in section 2.2.5. R848 encapsulation was determined by dissolving PAL-NPs particles in DMSO (Tocris, Cat# 3176), followed by absorbance readings against a R848 standard curve at 324 nm. PUUC RNA loading was quantified by Ribogreen assay according to the manufacturer’s instructions. CpG DNA loading was quantified by measurement of unbound DNA in the supernatant after centrifugation at 20,000 × g, using a Nucleic Acid Quantification workflow on a Synergy H.T. plate reader (BioTek) with Gen5 software.

**2.2.5. Nanoparticle’s stability**

A time-dependent PAL-NPs degradability behavior was evaluated using DTT as a reducing agent. The PAL-NPs (0.5 mg mL^-1^) dispersion with and without disulfide bond was prepared in PBS (10 mM, pH 7.4). The reducing agent dithiothreitol was added to the solution with the final concentration of 10 mM. The samples were incubated at 37 °C and protected from light. At regular time points (0 h, 2 h, 6 h, and 12 h), the particle’s average size was measured by DLS (Dynamic Light Scattering). Particle size degradation with respect to the time was plotted. (See **Fig S1c**).

**2.2.6. Transmission electronic microscopy (TEM)**

TEM was performed on a FEI Tecnai G2 F30 S-TWIN Transmission Electron Microscope at 300 kV. The 10 μL PAL-NPs solution (10 times a diluted sample of 0.5 mg mL^-1^) was placed on the copper grids for sample preparation. The excess solution was absorbed by Whatman filter paper at the edges and dried for 10 sec at RT. Samples were further stained by a drop of phosphotungstic acid (stock solution of 2%) onto the surface of the sample-loaded grid. The grid was washed twice with DI water to remove excess staining reagent. Grids were dried in desiccators overnight and analyzed by transmission electron microscopy.

**2.2.7. Adjuvant release study from PAL-NPs**

The PUUC PAL-NPs (NPs: 5 mg mL^-1^, PUUC: 100 ug) dispersion was prepared in PBS (10 mM, pH 7.4). For low pH solutions, the PAL-NPs (5 mg mL^-1^) dispersion was prepared in PBS (10 mM, pH 5.5). For a combination of low pH and the presence of the reducing agent dithiothreitol, the pH was raised to pH 7.4 before the addition of DTT. The PAL-NPs were incubated at low pH, with DTT for 8h in an Eppendorf tube. After 8 hours, the particles were centrifuged at 20,000g for 10 minutes. The supernatant was collected, and PUUC RNA was isolated using 30KDa amicon filters. The extracted PUUC RNA loading was quantified by Ribogreen assay according to the manufacturer’s instructions. (See **Fig S1E**).

**2.3. Immunological studies:**

**2.3.1.** **In vitro activation of mouse BMDCs with combination adjuvants of PAL-NPs formulations**

GM-CSF-derived BMDCs were generated following previously published procedures (*4*). At day 7 of the culture, mBMDCs derived from GM-CSF were seeded in 96-well plates at a density of 500 × 10^3^ cells per well and allowed to settle for 2 h. Adjuvanted PAL-NP formulations (see Table S1 for adjuvant and PAL-NP doses) were then added to the wells. After treatment, the supernatants were collected 24 hours later, and cytokine concentrations (IL-1β, IFN-β, and IL12p70) were measured using ELISA assays.

**2.3.2.** **Euthanasia and sample collection (BAL fluid, blood, and lungs)**

For IM and IN in vivo studies, mice were euthanized at day 35 (after 2 weeks of booster dose), and blood, BAL fluid, and lungs were harvested. Mice were initially anesthetized using an optimized mixture of ketamine (80 mg kg^-1^) and xylazine (15 mg kg^-1^), injected 25 µl intraperitoneally first and 50 ul intramuscularly later (7-8 minutes later). Blood was first collected from all mice via the jugular veins. All blood samples were allowed to clot for 30–60 min at RT in serum separator tubes (B.D., #365967), and serum was separated by centrifugation at 4000 x g for 15 min at 4 °C. Serum samples were heat-inactivated at 56 °C for 30 min in a water bath, which inhibits the complement binding. After inactivation, serum samples were aliquoted and stored at -80 °C. BAL fluid was collected after two separate injections and withdrawals (total 2 ml in Hanks' Balanced Salt Solution, sigma Aldrich cat#H4641 with 100 µM EDTA Sigma Aldrich cat#03699,) by inserting a 20 gauze one-inch catheter into the trachea following the methods described in Hoecke et al *(4)*. Samples were centrifuged at 300 x g for 5 minutes to remove cells. BAL Samples were further concentrated 10x using 100 KDa Amicon concentrators and aliquoted and stored at -80 °C.

**2.3.3.** **Ex vivo lung cell restimulation and T cell staining**

After harvesting the lungs from vaccinated mice (individual experiment), single-cell suspensions were prepared with gentleMACS™ Octo Dissociator and Lung Dissociation Kit (Miltenyi Biotec) according to the manufacturer’s instructions, including RBC lysis. Cells were centrifuged and resuspended at 10 million cells mL^-1^ in RPMI media with 10% FBS, 1% penicillin-streptomycin, 1 mM sodium pyruvate, and 1x β-mercaptoethanol. Cells were seeded at 2 million cells per well in a U-bottom 96-well plate and left to culture overnight (at 37 °C with 5% CO_2_). Lung cells were centrifuged and resuspended with fresh complete RPMI media with 20 μL mL^-1^ of PeptTivator® SARS-CoV-2 Prot_S (Miltenyi Biotec) and 5 μg mL^-1^ Brefeldin A (Biolegend). After incubation for 6 h, cells were stained for 30 min at RT with Zombie Green™ Fixable Viability Kit (Biolegend) and were blocked with anti-mouse CD16/32 (Biolegend) and True-Stain Monocyte Blocker™ (Biolegend). For blocking of cell surfaces, the cells were stained for 30 min at 4 °C with surface antibodies: anti-mouse CD3 (Biolegend, APC Fire 810), CD4 (Biolegend, APC), CD8a (Biolegend, PE/Cy5), CD44 (Biolegend, BV711), CD69 (Biolegend, BV785), CD103 (Biolegend, PE-Dazzle 594), CD56 (BD, BUV395), and TCR-γδ (Biolegend BV510). After surface staining, the cells were stained for intracellular cytokines. The cells were fixed and permeabilized for 30 min with the Foxp3/Transcription Factor Staining Buffer Set (eBioscience) at 4 °C. Then, cells were stained with anti-mouse TNF-α (Biolegend, PE/Cy7), IFN-γ (Biolegend, PE), and Granzyme B (Biolegend, Pacific Blue). Cell population data were acquired on Cytek Aurora flow cytometer and analyzed using FlowJo Software (**Fig. S8A** for the gating strategy). Fold changes in T cell response were evaluated with respect to PBS.

**2.3.4.** **B cell staining and flow cytometry**

Lung single-cell suspensions were stained for 30 min at RT with Zombie Red™ Fixable Viability Kit (BioLegend) and were blocked with anti-mouse CD16/32 (Biolegend) and True-Stain Monocyte Blocker™ (Biolegend). Cells were washed once with PBS before surface staining. Following blocking, cell surfaces were stained for 30 min at 4 °C with anti-mouse GL7 (Biolegend, Pacific Blue), IgM (Biolegend, Pe-Cy7), CD138 (Biolegend, PerCP/Cy5.5), CD19 (Biolegend, AF700), IgA (SouthernBiotech, FITC), B220 (Biolegend, BV711), CD38 (Biolegend, APC Fire 750), and anti-IgD (Biolegend, BV605), PE-SARS-CoV-2 RBD tetramer, APC-SARS-CoV-2 RBD tetramer for 30 min at 4 °C (tetramer synthesis was described in section 2.3.5). After washing with PBS, cells were fixed using 4% paraformaldehyde. Cell population data were acquired on Cytek Aurora flow cytometer and analyzed using FlowJo Software (**Fig. S8B** for gating strategy).

**2.3.5. SARS-CoV-2 RBD B-cell tetramer synthesis**

RBD tetramer was prepared by a previously published procedure *(5)*. Recombinant Biotinylated SARS-CoV-2 S protein RBD, His, Avitag™ (ACRO Biosystems SPD-C82E9) was incubated at a 4:1 molar ratio with either streptavidin-PE (Biolegend, 405204) or streptavidin- APC (Biolegend, 405207) in PEB buffer (1X PBS + 0.5% BSA 2 mM EDTA) for one hour at 4 °C. The mixture was then purified, concentrated in an Amicon Ultra (50 kDa MWCO) spin column, and washed with sterile, cold PBS. Excess streptavidin was blocked with biotin. Final protein concentration was measured on a nanodrop, using a protein quantification workflow on a Synergy H.T. plate reader (BioTek) with Gen5 software. Tetramers were diluted to 1.0 μM in PBS and stored at 4 °C.

**2.3.6. Supernatant cytokine profile**

Supernatants from the lung T cell restimulation assay were harvested, and T_H_1/T_H_2 cytokine production was measured using LEGENDplex™ (Mouse T_H_1/T_H_2 Panel, Biolegend, 741054) for IL-5, IL-13, IL-2, IL-6, IL-10, IFN-γ, TNF-α, IL-4, according to manufacturer's instructions. Cytokine beads were analyzed on a cytoflex flow cytometer. Raw data were analyzed using LegendPlex software (Biolegend), and the average cytokine level was determined from two duplicate samples.

**2.3.7.** **ELISA assay for quantifying anti-spike antibody responses**

The diluted recombinant SARS-CoV-2 Spike His Protein, CF (R&D Systems, Cat# 11058-CV) (1 μg mL^-1^ in 0.05 M carbonate-bicarbonate buffer, pH 9.6) was coated onto Nunc™ MaxiSorp™ ELISA plates by adding 100 ng/well and incubating the plates overnight at 4 °C. Antigen-coated plates were washed three times with PBST wash buffer (prepared by mixing 10 mM PBS and 0.05% Tween-20 solution), and plates were blocked for six hours at 4°C with PBSTBA (prepared by mixing PBST with 1% BSA and 0.02% NaN_3_). Blocked plates were incubated overnight at 4 °C with diluted serum and BAL fluid samples (individual experiments). Plates were washed three times with PBST. A secondary biotinylated anti-mouse IgA, total IgG, IgG1, or IgG2a antibody (SouthernBiotech), which is 5,000-fold diluted in 5-fold diluted PBSTBA, was added to plates for 2 h at RT. Plates were similarly washed with PBST. After two hours, a 5,000-fold diluted streptavidin-conjugated horseradish peroxidase (strep-HRP, ThermoFisher) was added to the plates and incubated for the next 2 h at RT. The plates were again washed six times. Ultra TMB-ELISA Substrate Solution (ThermoFisher) was incubated for 15 to 20 minutes for color development on the plate. Lastly, stop solution (2 N sulfuric acid) was added to each well, and absorbance was measured at 450 and 630 nm (background) on a Synergy H.T. plate reader (BioTek) with Gen5 software. In ELISA-based antibody titer quantifications, titers were determined by identifying the highest fold dilution of the sample that produced an absorbance reading above a predetermined cutoff value. Specifically, for total IgG, IgG1, and IgG2a titers, the cutoff value was established as the mean absorbance plus three times the sample standard deviation of the absorbance values obtained from the least-diluted PBS control groups.

**2.3.8.** **Modified ELISA assay to measure anti-spike neutralizing antibodies**

The above-described ELISA method was used with slight modification to quantify neutralizing antibodies. A diluted recombinant SARS-CoV-2 Spike His Protein, CF (R&D Systems, Cat# 11058-CV) in 0.05 M carbonate-bicarbonate buffer (1 μg spike protein mL^-1^, pH 9.6) was incubated in wells of a Nunc™ MaxiSorp™ ELISA 96-well plate (100 ng/well) overnight at 4 °C. Plates were washed three times with PBS-Tween wash buffer (PBST). Antigen-coated plates were blocked in PBSTBA for six hours at 4 °C. Blocked plates were again incubated overnight with serum and BAL fluid samples (individual experiments). Plates were similarly washed. Plates were incubated with PBSTBA diluted 500 ng mL^-1^ (25 ng/well) recombinant biotinylated human ACE-2 (R&D Systems, Cat# BT933-020) for 2 h at RT. The plates were washed again with PBST. A 5,000-fold diluted strep-HRP (ThermoFisher) was added to the plates and incubated for 2 h at RT. Plates were extensively washed six times and incubated with the optimized volume of Ultra TMB-ELISA Substrate Solution (ThermoFisher) for 20 min. In the end, the reaction was stopped with a stopping reagent (2 N sulfuric acid), and absorbance was measured at 450 and 630 nm (background) on a Synergy H.T. plate reader (BioTek) with Gen5 software.

**3. Figures**

**
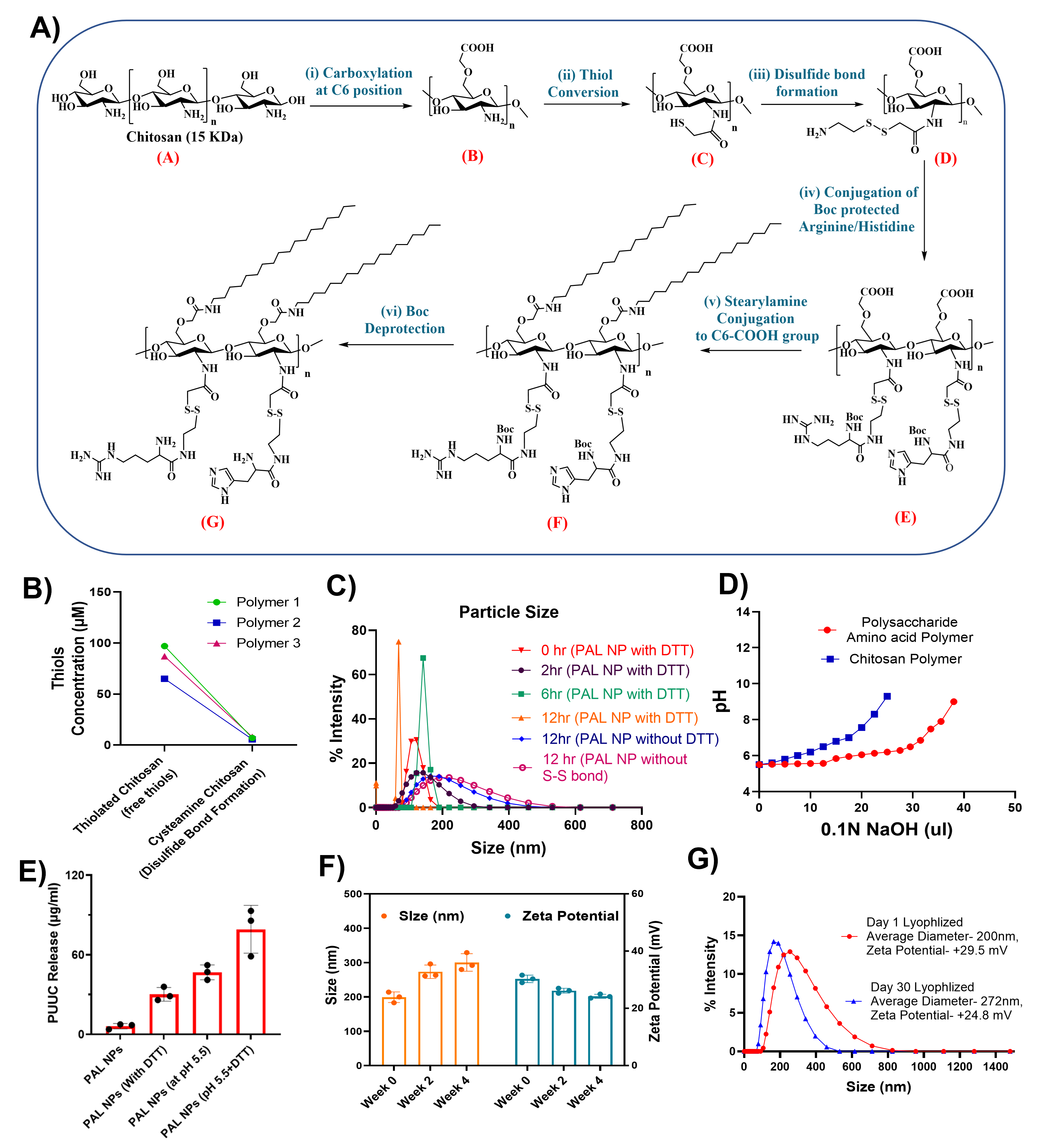
Figure S1**. Synthetic scheme of PAL polymer and PAL-NPs characterization. (A) Multistep synthesis of polysaccharide-amino acid-lipid amphiphilic (PAL) polymer- (i) Chitosan, NaOH, -10 ^o^C incubation, 1 h, Cl-CH_2_-COOH, heat (45 ^o^C), 24 h (ii) EDC/NHS, HS-CH_2_COOH (iii) NH_2-_CH_2_-CH_2_-SH, cysteamine, pH= 6 (iv) EDC/NHS, Nα-Boc-L-arginine and Nα-Boc-L-histidine (v) EDC/NHS, CH_3_(CH_2_)_17_NH_2_, heating 80 ^o^C (vi) TFA/4 M HCl in Dioxane, Boc deprotection. (B) Estimation of thiols and disulfide concentration in thiolated polymer and cysteamine conjugated chitosan polymer by Elmann assay. (C) Time-dependent degradation study of the PAL-NPs by DLS analysis in the presence of DTT (10 mM). D) Buffering capacity of Polysaccharide Amino Acid Polymer (without lipid) and comparison with chitosan polymer, E) PUUC adjuvant release from PUUC PAL-NPs in the presence of DTT, at low pH, and in a combination of low pH and with DTT. F) Size and zeta potential of PAL-NPs over four weeks in PBS, G) Size and zeta potential of PAL-NPs after lyophilization at Day 1 and Day 30.


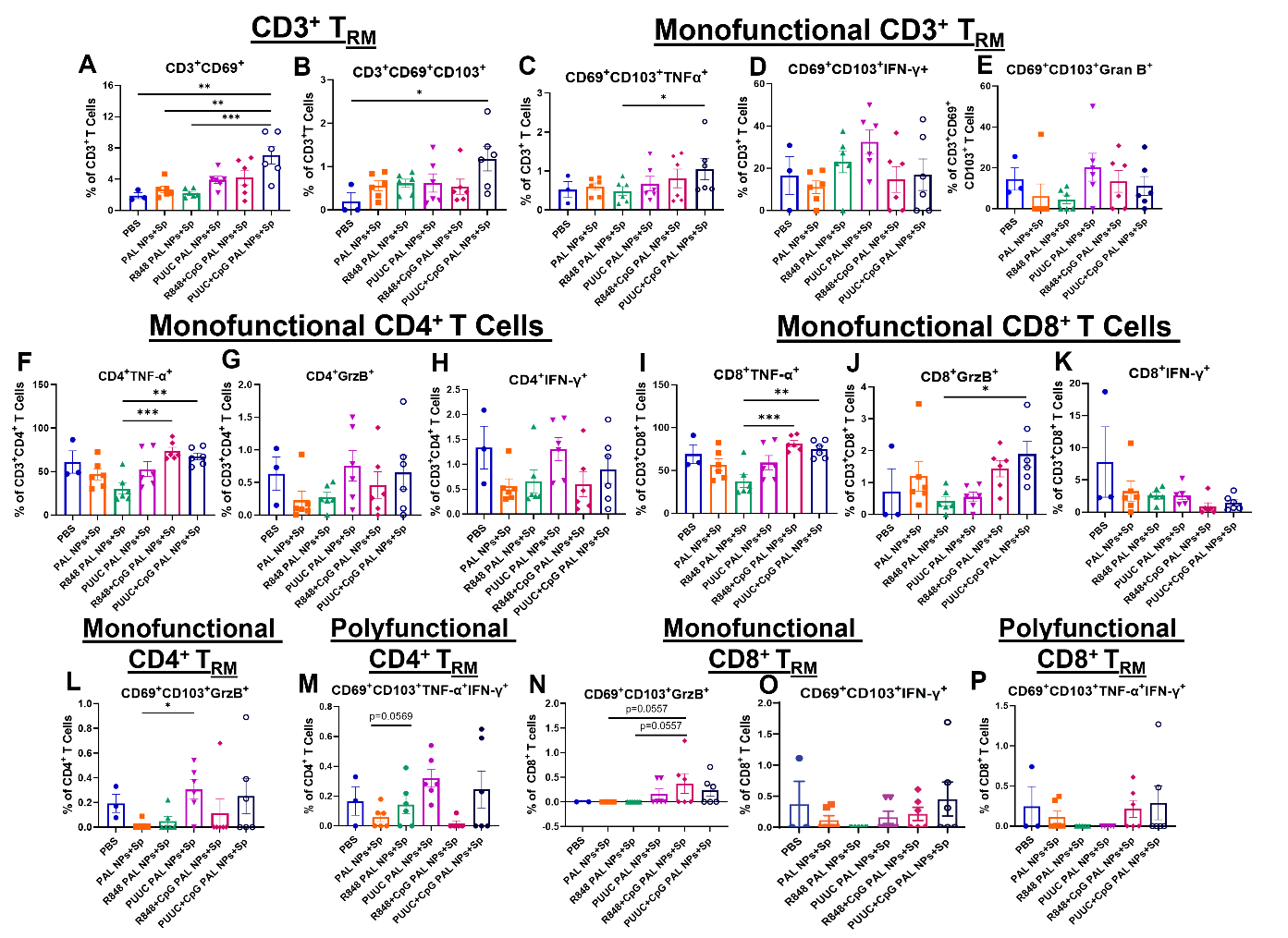


**Figure S2.** PUUC+CpG PAL subunit nanovaccine formulation with S1 spike protein, elicits robust SARS-CoV-2 elicits T cell immunity when delivered IM-Prime/IN-Boost. On days 0 (IM prime) and 21 (IN boost), female BALB/c mice were immunized with adjuvanted PAL-NP vaccine formulation with S1 spike protein (see Materials/Methods and Table 1 for doses). Mice were euthanized, and lungs were collected on Day 35 (one-week post-boost). Lung cells were restimulated with spike peptide for 6 h. (A and B) Percentage of CD3^+^CD69^+^CD103^-^ and CD3^+^CD69^+^CD103^+^ (CD3^+^ T_RM_) cell population. (C to E) Percentage of monofunctional CD3^+^ T_RM_ cells expressing TNF-α, IFN-γ, and GrzB. (F to H) Percentage of monofunctional CD4^+^ T cells expressing TNF-α, IFN-γ, and GrzB. (I to K) Percentage of monofunctional CD8^+^ T cells expressing TNF-α, IFN-γ, and GrzB. (L) Percentage of monofunctional CD4^+^ T_RM_ cells expressing GrzB. (M) Percentage of polyfunctional CD4^+^ T_RM_ cells co-expressing TNF-α, and IFN-γ. (N) Percentage of monofunctional CD8^+^ T_RM_ cells expressing GrzB. (O) Percentage of monofunctional CD8^+^ T_RM_ cells expressing IFN-γ. (P) Percentage of polyfunctional CD8^+^ T_RM_ cells co-expressing TNF-α, IFN-γ. Error bars represent the SEM. Statistical significance was calculated using one-way ANOVA followed by Tukey’s post-hoc test for the figures (A), (B), (J), and (K), and Bonferroni's post-hoc test for the figures (C), (I), and (M), for multiple comparisons. **p* ≤ 0.05, ***p* ≤ 0.01, ****p* ≤ 0.001, *****p* ≤ 0.0001 for all graphs.


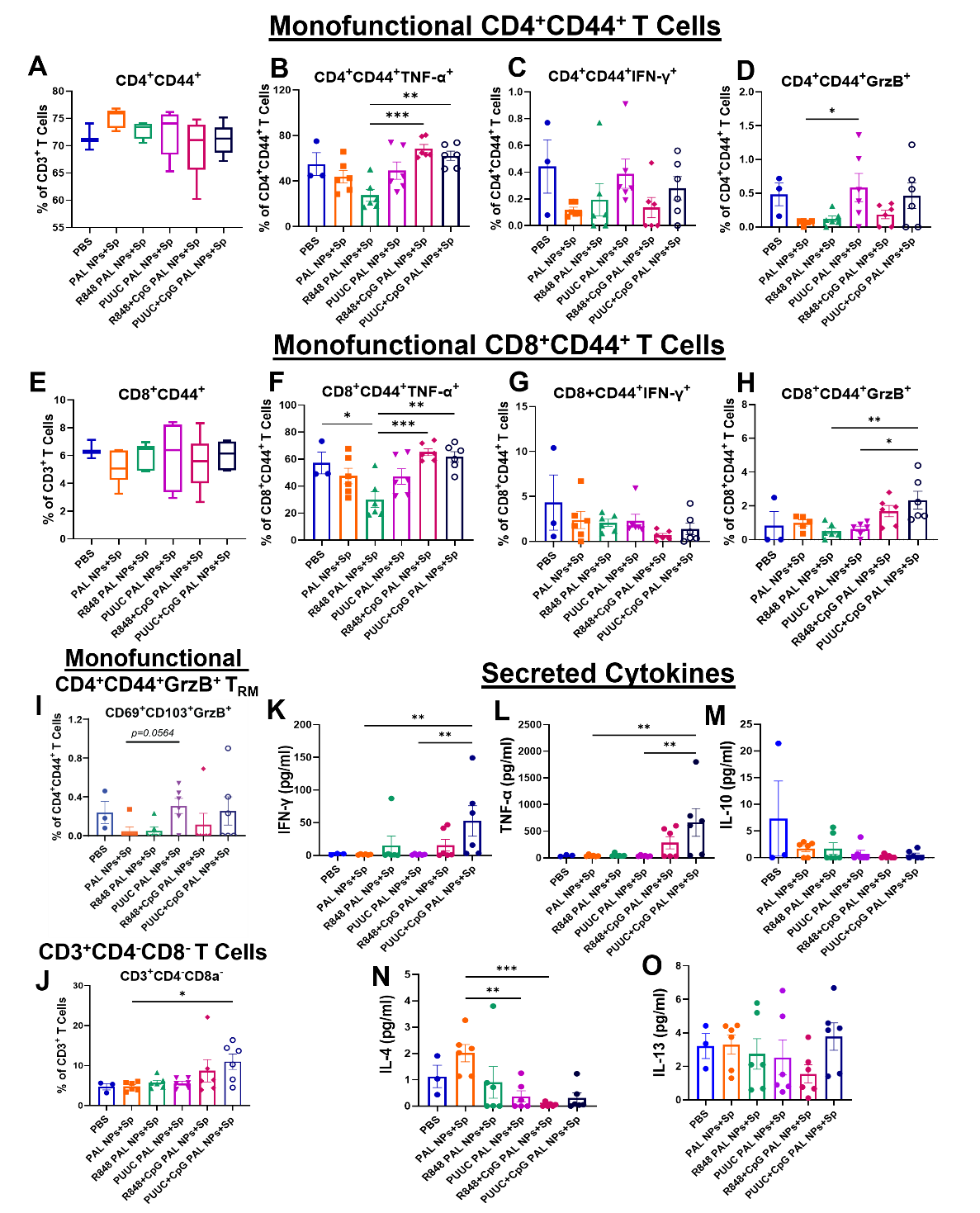


**Figure S3.** PUUC+CpG PAL subunit nanovaccine formulation with S1 spike protein, elicits robust SARS-CoV-2 elicits T cell immunity when delivered IM-Prime/IN-Boost. On days 0 (IM prime) and 21 (IN boost), female BALB/c mice (n=3 for PBS and n=6 for other adjuvanted PAL-NP groups) were immunized with adjuvanted PAL-NP vaccine formulation with S1 spike protein (see Materials/Methods and Table 1 for doses). Mice were euthanized, and lungs were collected on Day 35 (one-week post-boost). Lung cells were restimulated with spike peptide for 6 h. (A) Percentage of CD4^+^CD44^+^ cell population. (B to D) Percentage of CD4^+^CD44^+^ cells expressing TNF-α, IFN-γ, and GrzB. (E) Percentage of monofunctional cells expressing CD8^+^CD44^+^. (F to H) Percentage of monofunctional CD8^+^CD44^+^ T cells expressing TNF-α, IFN-γ, and GrzB. (I) Percentage of monofunctional CD4^+^CD44^+^ T_RM_ cells expressing GrzB. (J) Percentage of CD3^+^CD4^-^CD8^-^ cell population. (K to P) Cytokine concentration TNF-α, IFN-γ, IL-10, IL-4, IL13, IL12p40 in supernatants from restimulated lung cells. Error bars represent the SEM. Statistical significance was calculated using one-way ANOVA followed by Tukey’s post-hoc test for the figures (B), (F), and (H), and Bonferroni's post-hoc test for the figures (D), (I), and (K), for multiple comparisons. Statistical significance for cytokine concentrations was calculated with One-Way ANOVA and Tukey post-hoc test. **p* ≤ 0.05, ***p* ≤ 0.01, ****p* ≤ 0.001, *****p* ≤ 0.0001 for all graphs.

**
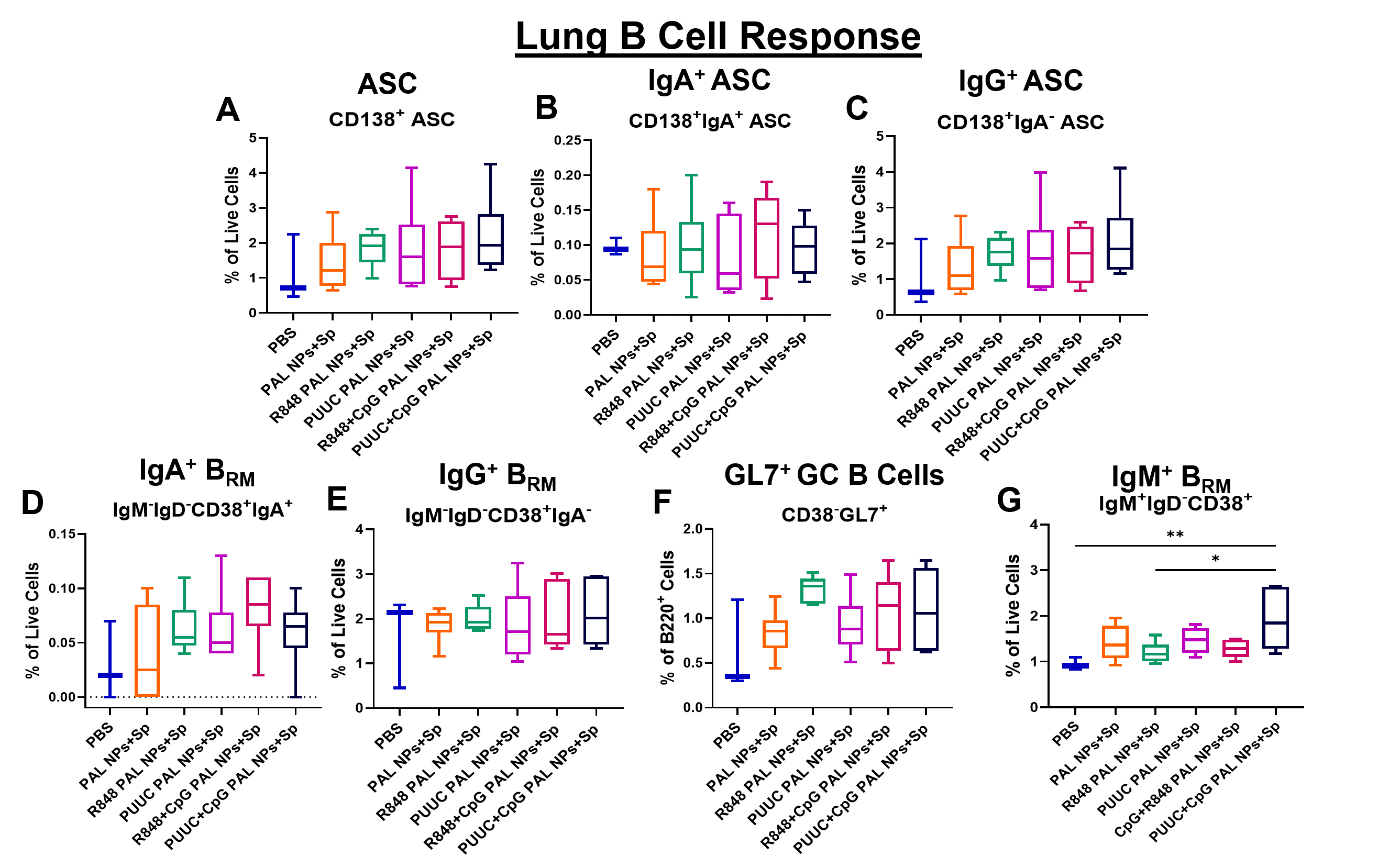
**

**Figure S4.** Analysis of lung B cell responses when adjuvanted PAL subunit nanovaccine formulations are delivered to mice via IM-Prime/IN-Boost vaccination. On days 0 (IM prime) and 21 (IN boost), female BALB/c mice (n=3 for PBS and n=6 for other PAL-NP groups) were immunized with adjuvanted PAL-NP vaccine formulation with S1 spike protein (see Materials/Methods and Table S1 for doses). Mice were euthanized, and lungs were collected on Day 35 (one-week post-boost). (A) Percentage of CD138^+^ASC population. (B) Percentage of IgA^+^ASC population. (C) Percentage of IgG^+^ASC population. (D) Percentage of IgA^+^B_RM_ cell population. (E) Percentage of IgG^+^B_RM_ cell population. (F) Percentage of GL7^+^ GC B cell population. (G) Percentage of IgM^+^ Memory B cell population. Error bars represent the SEM. Statistical significance was calculated with One-Way ANOVA and Tukey post-hoc test. **p* ≤ 0.05, ***p* ≤ 0.01, ****p* ≤ 0.001, *****p* ≤ 0.0001 for all graphs.

**
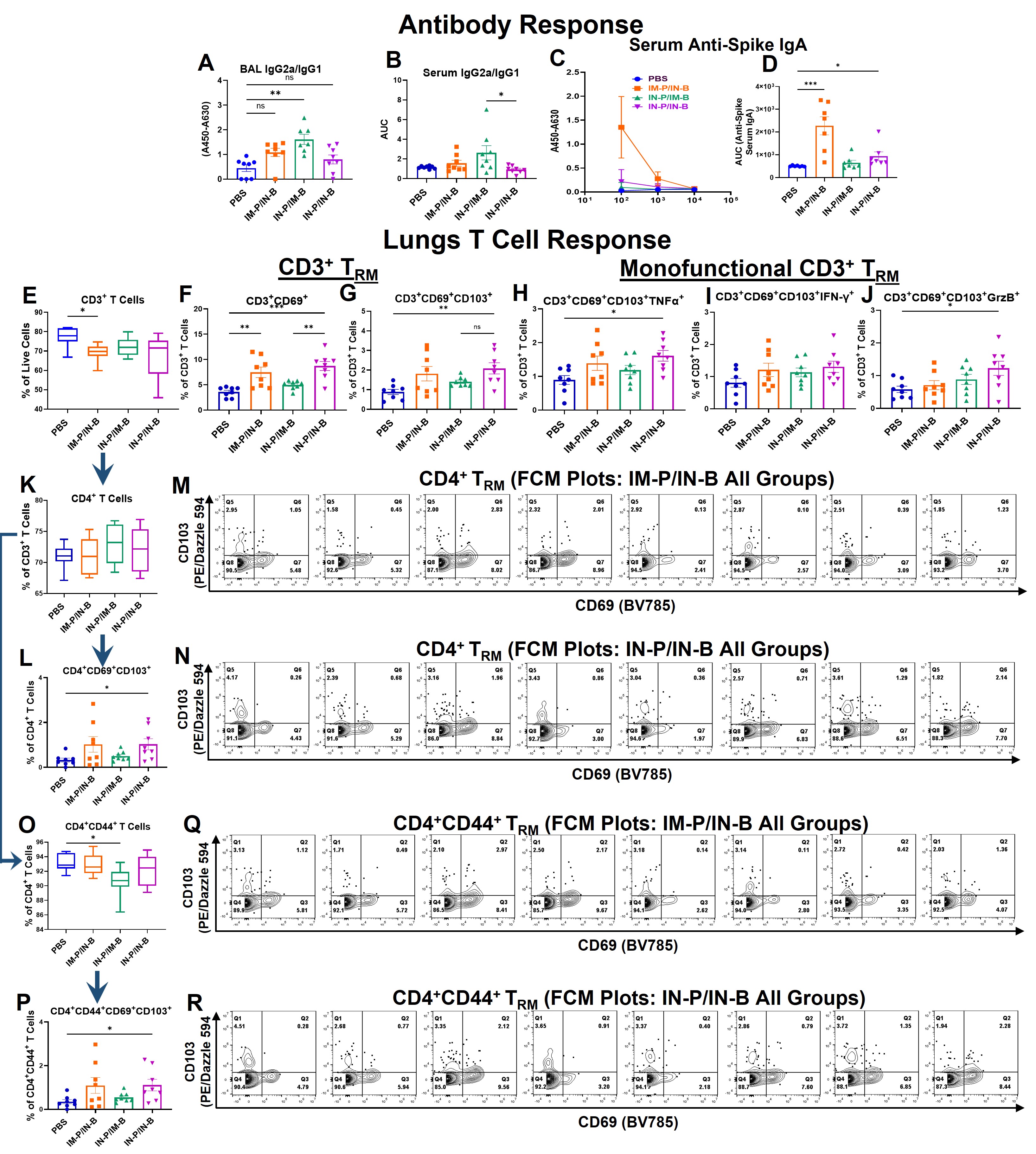
Figure S5.** PUUC+CpG PAL subunit nanovaccine formulation with S1 spike protein elicits robust SARS-CoV-2 lung-specific T cell immune response with IN-Prime/IN-Boost strategy. Female BALB/c mice were immunized with PUUC+CpG PAL-NP vaccine formulation with S1 spike protein (see Materials/Methods and Table S1 for doses). Female BALB/c mice (n=8 for all groups) were immunized with three prime-boost strategies: IM-Prime/IN-Boost, IN-Prime/IM-Boost, and IN-Prime/IN-Boost. Mice were euthanized, and lungs were collected on Day 35. Lung cells were restimulated with spike peptide for 6h. (A) BAL IgG2a/IgG1. (B) Serum IgG2a/IgG1. (C) Serum from vaccinated mice was assayed for IgA (D) Comparison AUC of serum anti-spike IgA. (E-G) Percentage of CD3^+^, CD3^+^CD69^+^CD103^-^ and CD3^+^CD69^+^CD103^+^ (CD3^+^ T_RM_) cell population. (H-J) Percentages of monofunctional CD4^+^ T cells expressing TNF-α, IFN-γ, and GrzB. (K and L) Percentage of CD4^+^, and CD4^+^CD69^+^CD103^+^ (CD4^+^ T_RM_) cell population. (M-N) FCM plots of CD4^+^ T_RM_ and CD44^+^ T_RM_ in all the IM-Prime/IN-Boost and IN-Prime/IN-Boost (n=8). (O and P) Percentage of CD4^+^CD44^+^ and CD4^+^CD44^+^CD69^+^CD103^+^ (CD4^+^CD44^+^ T_RM_) cell population. (Q and R) FCM plots of CD4^+^ T_RM_ and CD44^+^ T_RM_ in all the IM-Prime/IN-Boost and IN-Prime/IN-Boost (n=8). Error bars represent the SEM. Statistical significance was calculated using one-way ANOVA followed by Tukey’s post-hoc test for multiple comparisons. **p* ≤ 0.05, ***p* ≤ 0.01, ****p* ≤ 0.001, *****p* ≤ 0.0001 for all graphs.

**
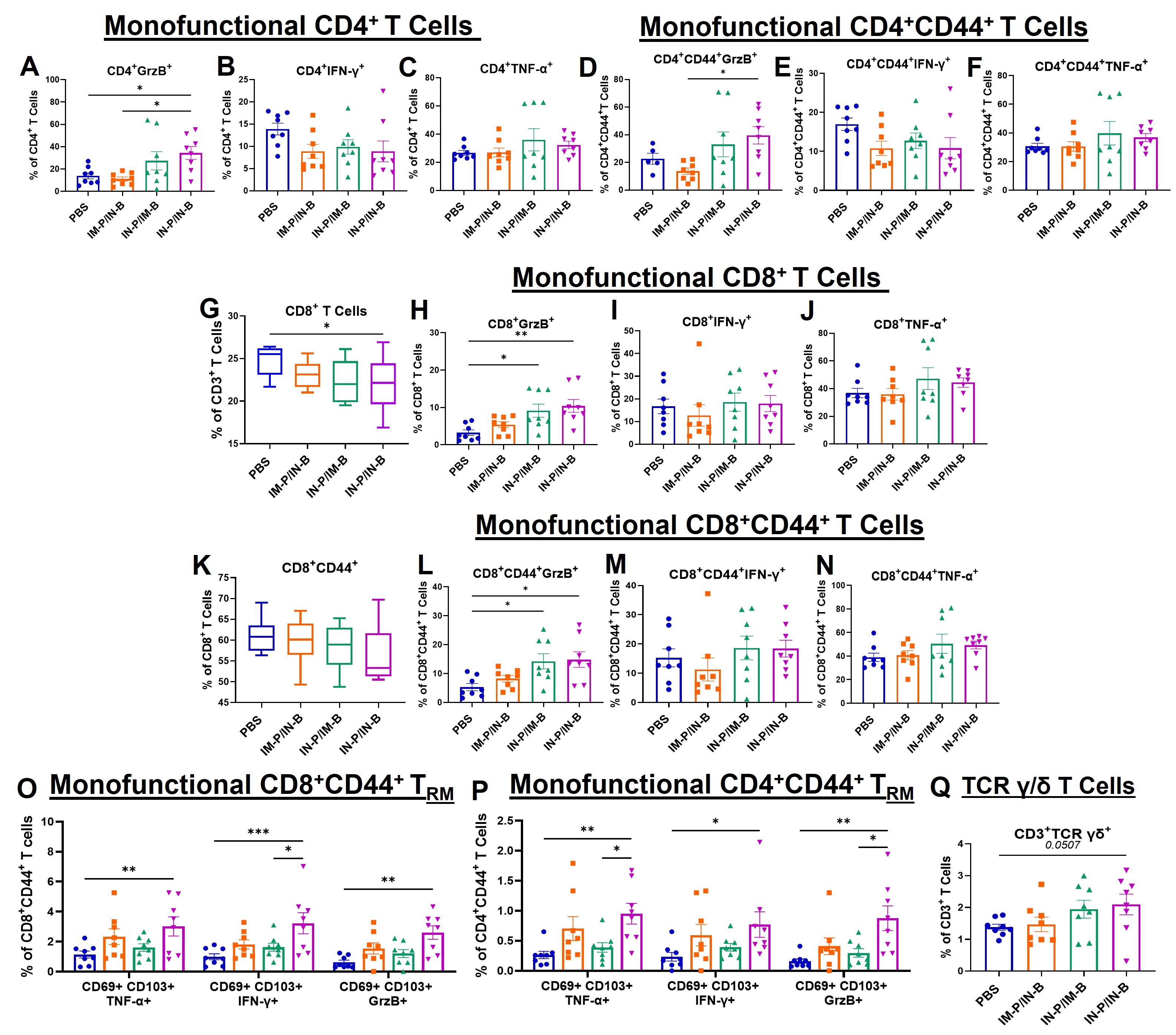
**

**Figure S6.** PUUC+CpG PAL subunit nanovaccine formulation with S1 spike protein, elicits robust SARS-CoV-2 T cell immune responses with IN-Prime/IN-Boost route. Female BALB/c mice were immunized with PUUC+CpG PAL-NP vaccine formulation with S1 spike protein (see Materials/Methods and Table S1 for doses). Female BALB/c mice (n=8 for all groups) were immunized with three prime-boost strategies: IM-Prime/IN-Boost, IN-Prime/IM-Boost, and IN-Prime/IN-Boost. Mice were euthanized, and lungs were collected on Day 35. Lung cells were restimulated with spike peptide for 6 h. (A-C) Percentages of CD4^+^ cell population expressing GrzB, IFN-γ, and TNFα. (D to F) Percentages of monofunctional CD4^+^CD44^+^ cells expressing GrzB, IFN-γ, and TNFα. (G) Percentage of CD8^+^ cell population. (H-J) Percentages of CD8^+^ cell population expressing GrzB, IFN-γ, and TNFα. (K) Percentage of CD8^+^CD44^+^ cell population. (L-N) Percentages of monofunctional CD8^+^CD44^+^ T cells expressing GrzB, IFN-γ, and TNFα. (O) Percentages of monofunctional CD4^+^CD44^+^ T_RM_ cells expressing TNFα, IFN-γ, and GrzB. (P) Percentages of monofunctional CD8^+^CD44^+^ T_RM_ cells expressing TNFα, IFN-γ, and GrzB. (Q) Percentages of CD3^+^ TCR γδ cells. Error bars represent the SEM. Statistical significance T cell frequencies were calculated with One-Way ANOVA and Tukey post-hoc test. **p* ≤ 0.05, ***p* ≤ 0.01, ****p* ≤ 0.001, *****p* ≤ 0.0001 for all graphs.


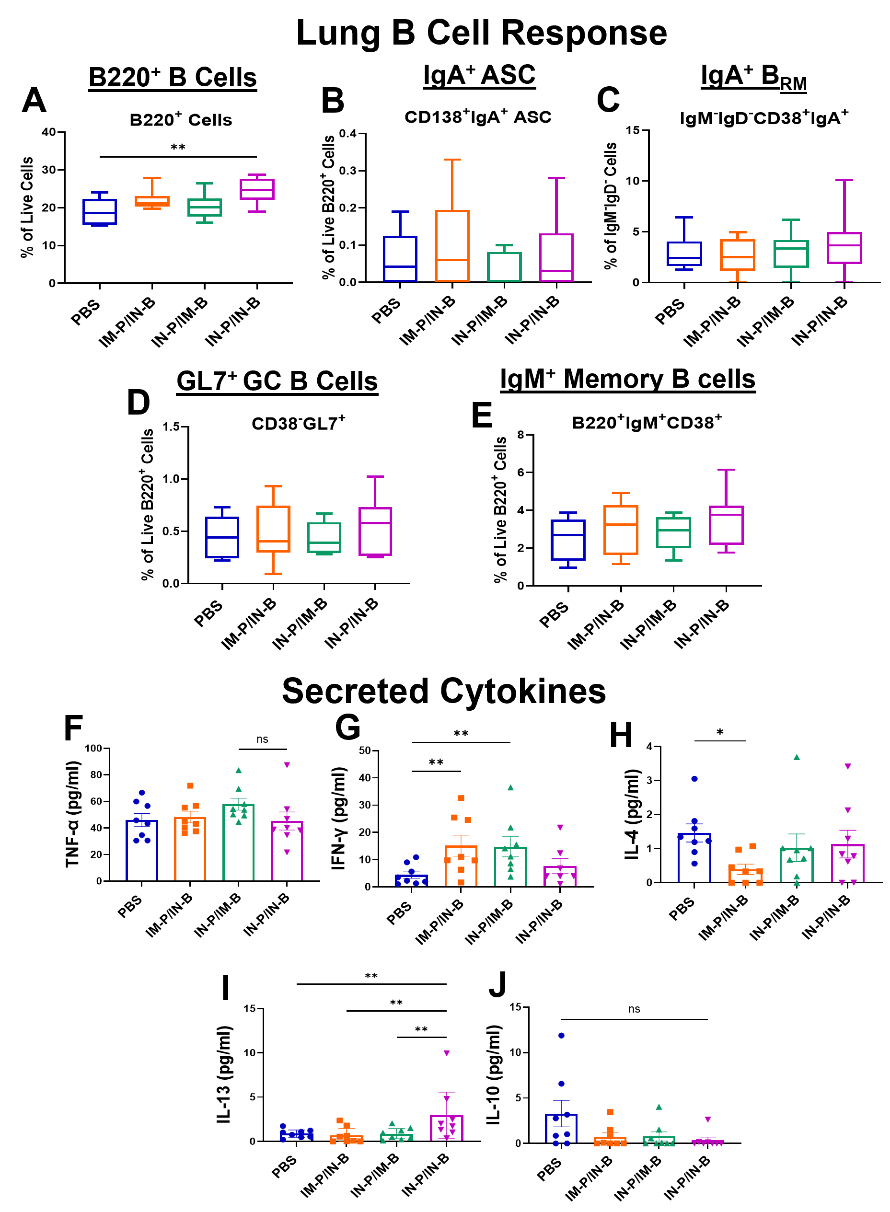


**Figure S7.** Lung-specific B cell and T cell (secreted cytokine) responses, when PUUC+CpG PAL PAL subunit nanovaccine, formulation and mixed with S1 spike protein, delivered with three different prime-boost routes. Female BALB/c mice were immunized with PUUC+CpG PAL-NP vaccine formulation with S1 spike protein (see Materials/Methods and Table S1 for doses). Female BALB/c mice (n=8 for all groups) were immunized with three prime-boost strategies: IM-Prime/IN-Boost, IN-Prime/IM-Boost, and IN-Prime/IN-Boost. On days 0 (prime) and 21 (boost), mice were euthanized, and lungs were collected on Day 35. Quantification of B cell response (A) Percentage of B220^+^ B cell population. (B) Percentage of IgA^+^ASC cell population. (C) Percentage of IgA^+^ B_RM_ cell population. (D) Percentage of GL7^+^ GC B cell population. (E) Percentage of IgM^+^ Memory B cell population. Lung cells were restimulated with spike peptide for 6 h. (F to J) Cytokine concentration in supernatants from restimulated lung cells: TNFα, IFN-γ, IL-4, IL-13, and IL-10. Error bars represent the SEM. Statistical significance T cell frequencies were calculated with One-Way ANOVA and Tukey post-hoc test. Statistical significance for cytokine concentrations was calculated with one-Way ANOVA and Tukey post-hoc test **p* ≤ 0.05, ***p* ≤ 0.01, ****p* ≤ 0.001, *****p* ≤ 0.0001 for all graphs. ns represents the non-significant values.

**
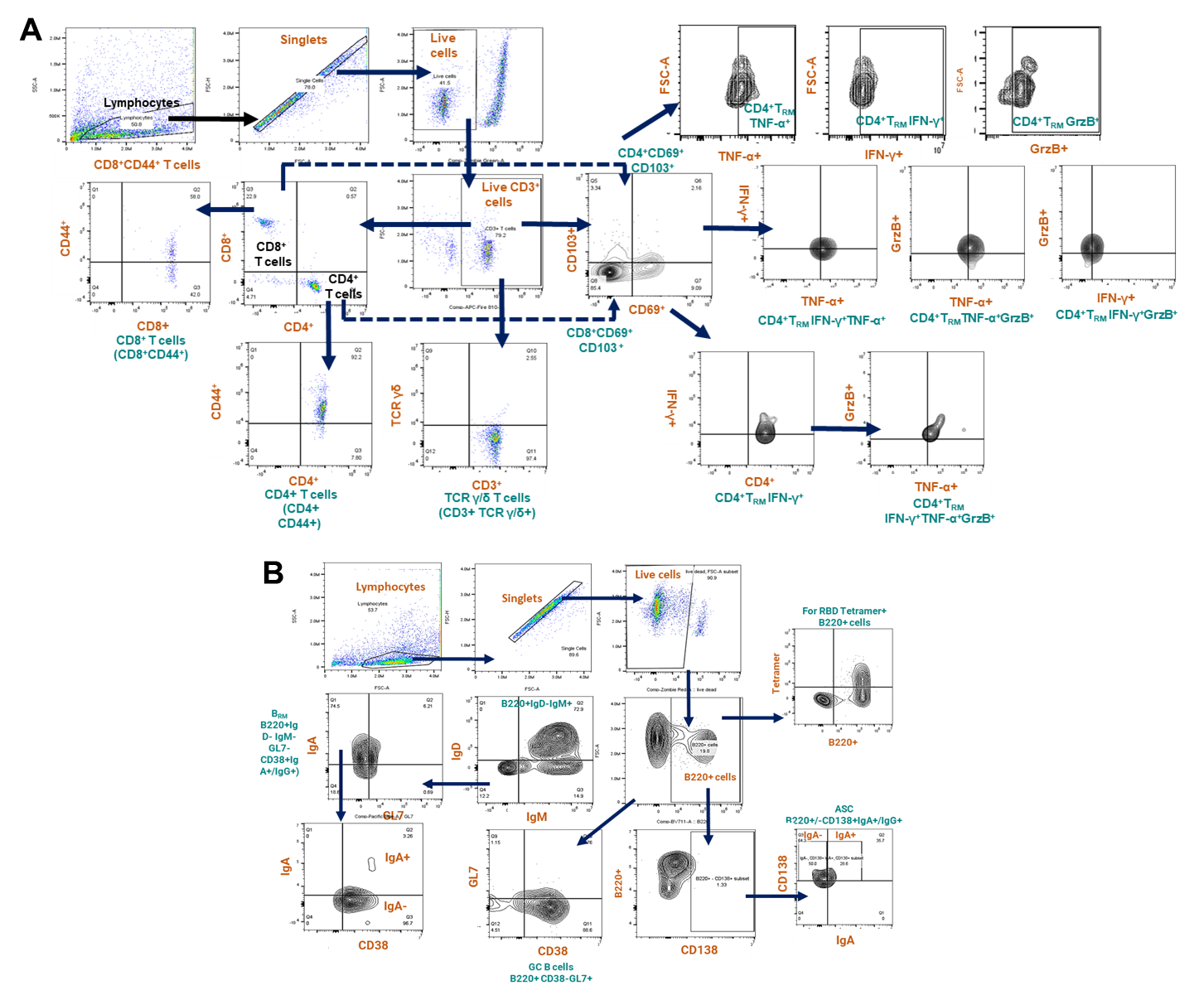
**

**Figure S8.** Gating strategies for analysis of adaptive immune responses in the lungs. (A) Gating strategies to identify CD4^+^ and CD8^+^ T cells and gating strategies to identify cytokine-producing CD4^+^ and CD8^+^ T cells. (B) Gating strategies to identify antigen-specific and polyclonal B cells.

**NMR Spectrum of Polymers**


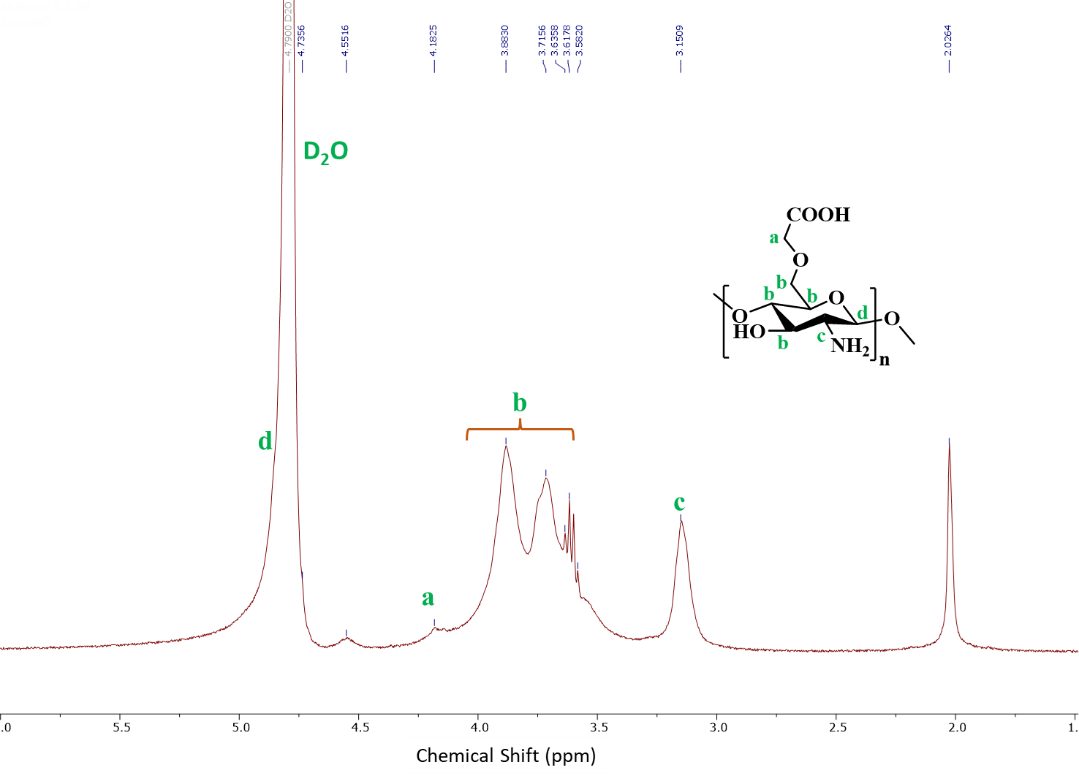


**Figure S9:** 400 MHz ^1^H NMR spectrum of the carboxylated chitosan (OCMC) in D_2_O with 1% DCl.


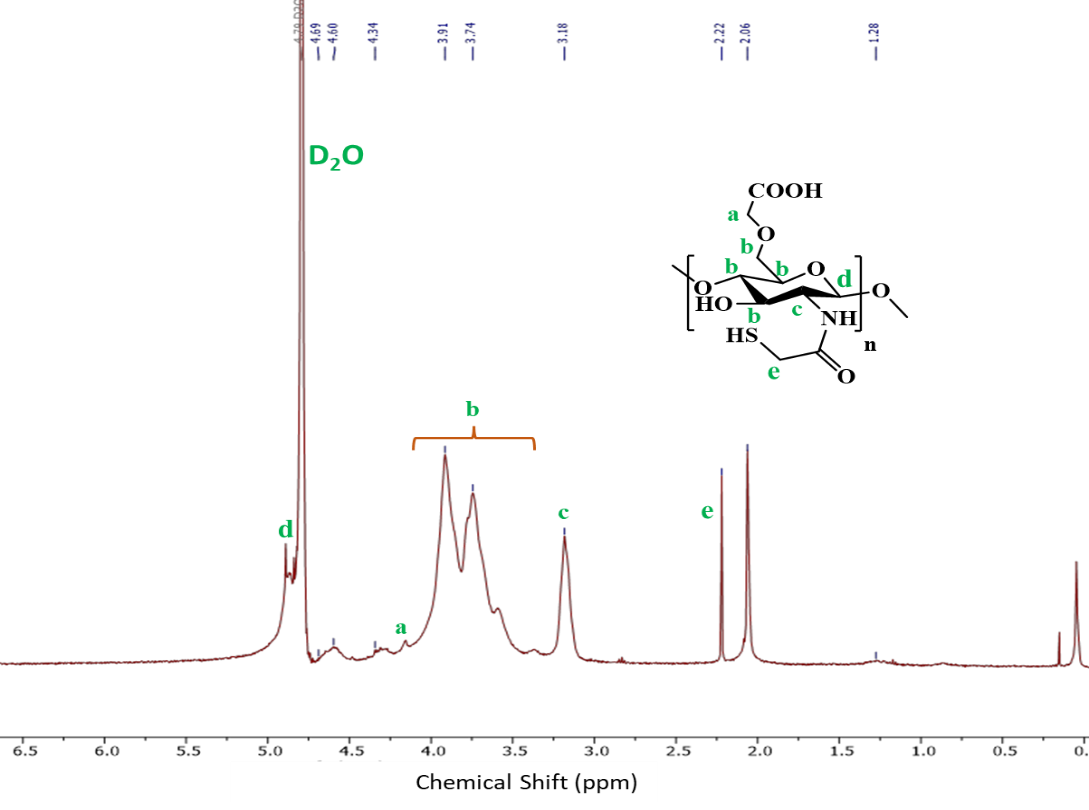


**Figure S10:** 400 MHz ^1^H NMR spectrum of the thiolated OCMC in D_2_O with 1% DCl.


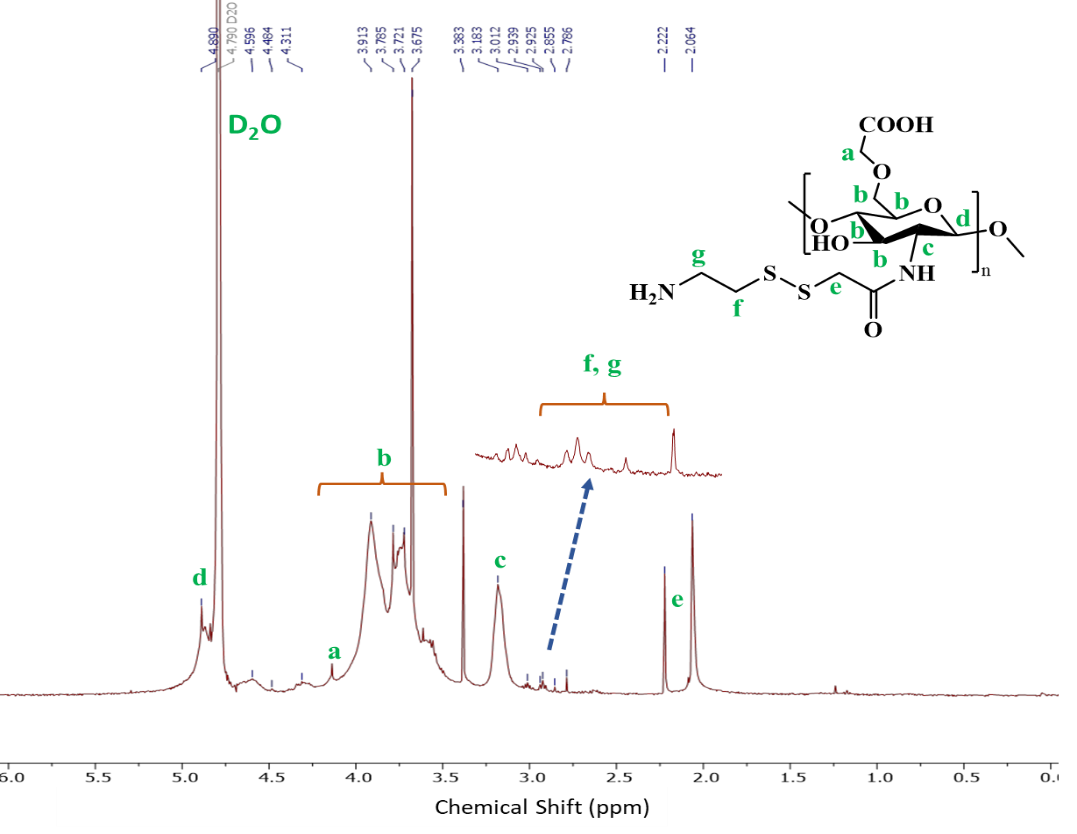


**Figure S11:** 400 MHz ^1^H NMR spectrum of the OCMC-S-S-Cys in D_2_O with 1% DCl.


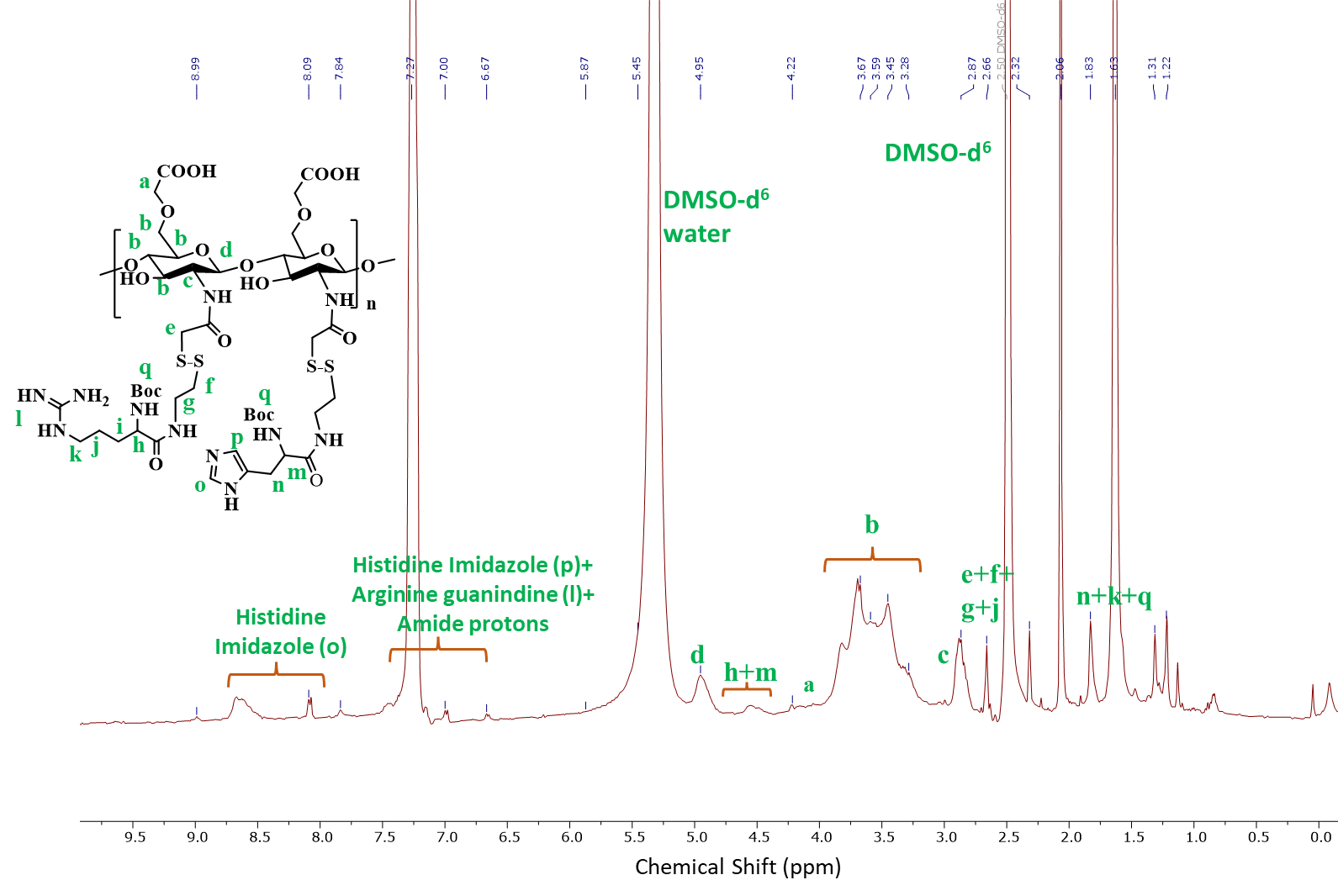


**Figure S12:** 400 MHz ^1^H NMR spectrum of the OCMC-S-S-(A/H) in DMSO-d^6^.


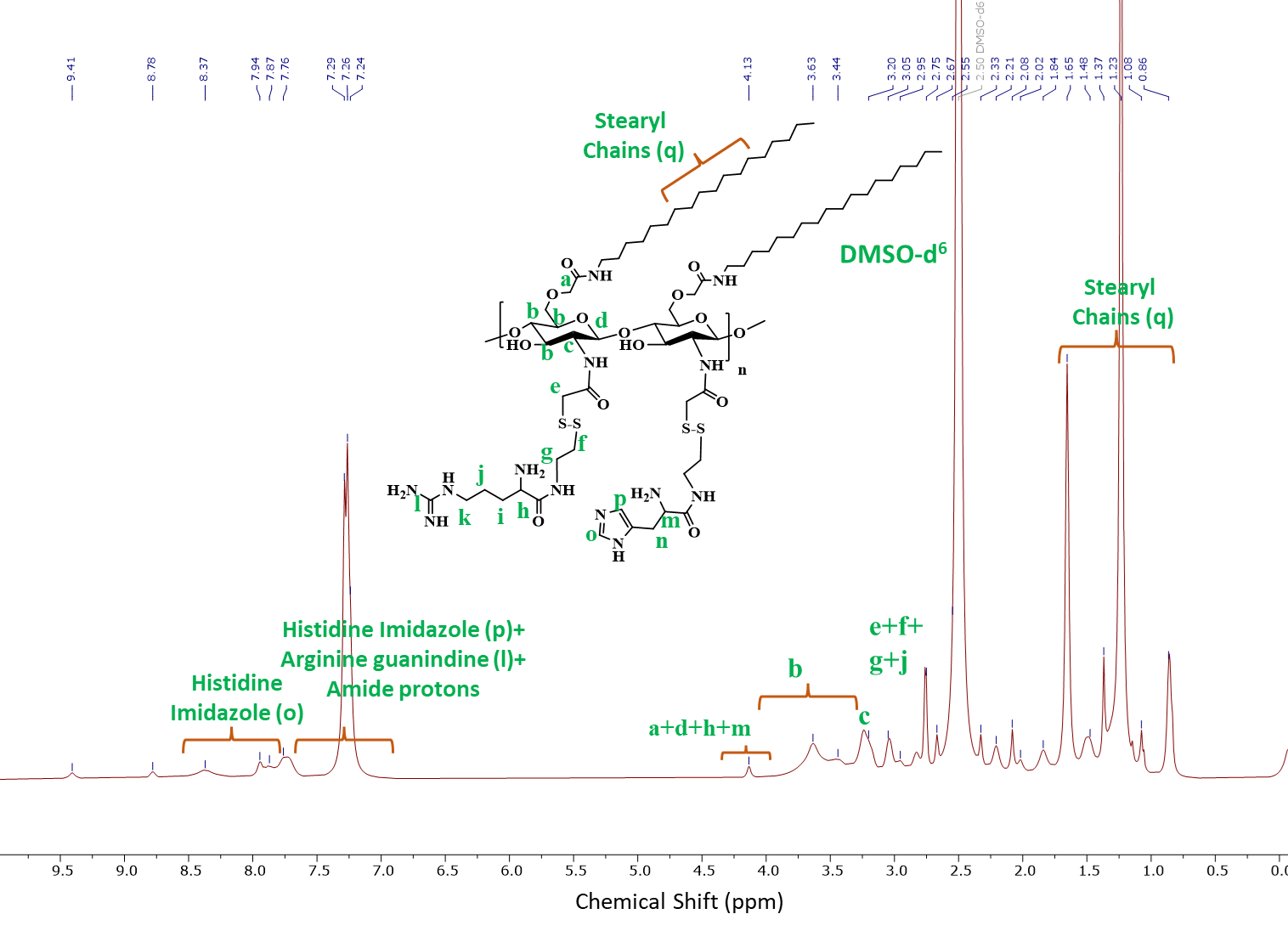
**Figure S13:** 400 MHz ^1^H NMR spectrum of the OCMC-S-S-(A/H)-SA in DMSO-d^6^.

**4. Table S1. Adjuvanted PAL-NPs formulations for in vitro and in vivo studies.**


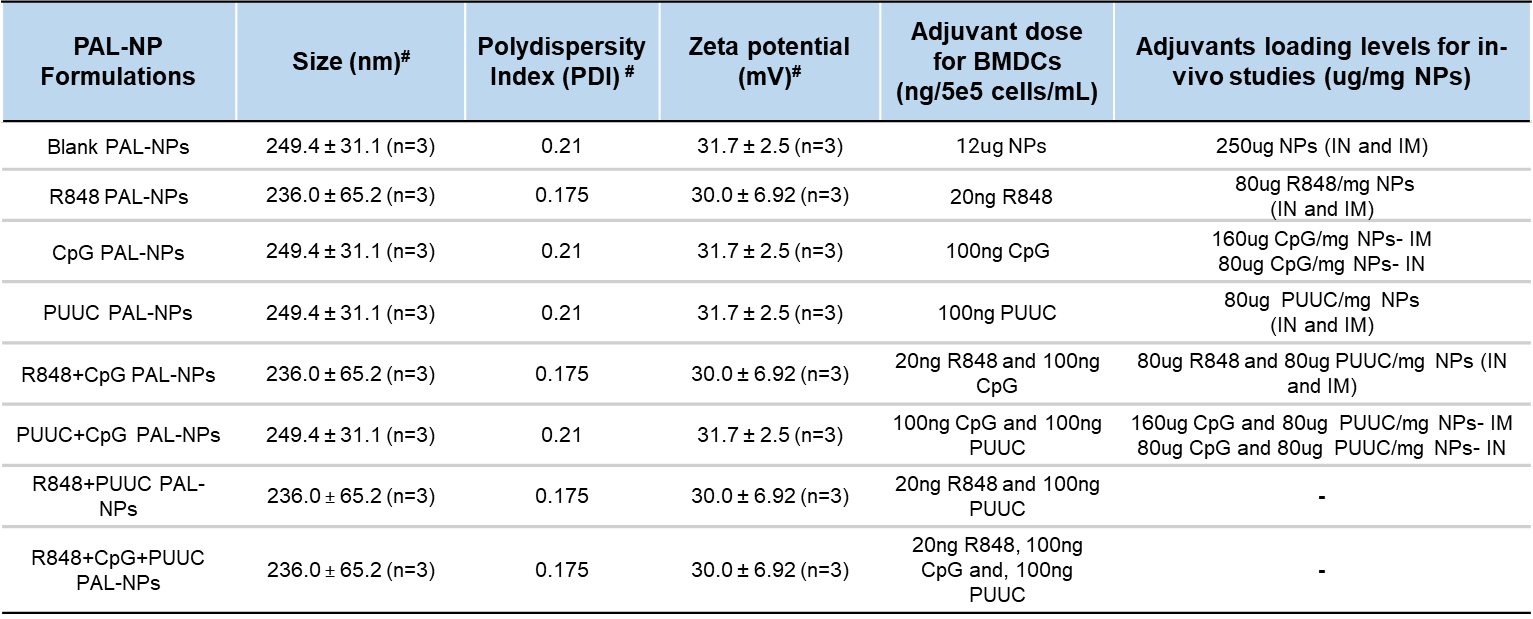
^#^Size, PDI, and zeta potential measurements were taken for all NPs prior to electrostatically loading adjuvants PUUC or CpG.

**5. References:**

[1] X. G. Chen, H. J. Park, Chemical characteristics of O-carboxymethyl chitosans related to the preparation conditions. *Carbohydrate Polymers*, **2003**, 53, 355-359.

[2] A. Bernkop-Schnürch, S. Steininger, Synthesis and characterization of mucoadhesive thiolated polymers. *International journal of pharmaceutics*, **2000**, 194, 239–247.

[3] A. Atalis, *et al.*, Nanoparticle-delivered TLR4 and RIG-I agonists enhance immune response to SARS-CoV-2 subunit vaccine. *Journal of Controlled Release* **2022,** 347, 476–488.

[4] L. V. Hoecke, *et al.*, Bronchoalveolar lavage of murine lungs to analyze inflammatory cell infiltration, *J Vis Exp*, **2017,** 123, e55398.

[5] T. Mao, *et al.*, Unadjuvanted intranasal spike vaccine elicits protective mucosal immunity against sarbecoviruses. *Science* **2022***,* 1979, **378**.
